# Effect of Functionally Selective Dopamine D_1_ Receptor Agonists on Complex Cognitive Processes in a Rodent Touchscreen Operant Chamber Task

**DOI:** 10.1523/ENEURO.0296-25.2026

**Published:** 2026-05-19

**Authors:** Ava P. Bassett, Luke Bransom, Richard B. Mailman, Yang Yang (杨杨)

**Affiliations:** ^1^Department of Neuroscience and Experimental Therapeutics, Penn State University College of Medicine, Hershey, Pennsylvania 17033; ^2^Department of Pharmacology, University of Virginia School of Medicine, Charlottesville, Virginia 22903

**Keywords:** dopamine D_1_ agonist, functional selectivity, touchscreen, TUNL, working memory

## Abstract

Dopamine D_1_ receptor (D_1_R) signaling in the brain has been strongly implicated in multiple cognitive processes, with D_1_ agonists known to enhance performance. The development of functionally selective D_1_ agonists that differentially activate D_1_R-mediated cAMP versus β-arrestin signaling may offer precision therapy if we understand how signaling bias impacts integrated cognitive processes in complex tasks. We therefore examined the effects of two selective D_1_ agonists, 2-methyldihydrexidine (2MDHX) and PF-06256142 (PF), on a rodent touchscreen-based Trial-Unique Nonmatching-To-Location task. Primarily assessing both spatial working memory and pattern separation in adult male rats, this behavioral paradigm requires greater cognitive demands to maintain performance throughout the testing session, significantly increasing task complexity. Our results revealed an inverted U-shaped dose response curve for both compounds, aligning with our previously published work, but did not demonstrate marked improvement in task performance. Subjects treated with the mid-dose range (10, 100, and 1,000 nmol/kg) generally performed similarly to, or slightly below, vehicle treated subjects whereas the highest dose administered (10,000 nmol/kg) significantly reduced task performance and engagement. The behavioral effects were similar for PF and 2MDHX, suggesting that functional selectivity/bias (at least for the cAMP versus β-arrestin signaling pathways) was not a major factor in this cognitive task. In addition to providing new information about how D_1_ agonists might affect different aspects of cognition, our data underscores the importance of dose optimization and task structure and showcases the translational value of touchscreen-based paradigms when assessing potential cognitive enhancers.

## Significance Statement

This study explored how functionally selective D_1_ receptor agonists may serve as potential cognitive enhancers. It highlighted the critical role of dose optimization in balancing efficacy and tolerability. By using a highly translational touchscreen-based task, these findings advance our understanding of how D_1_R signaling bias influences working memory and helps provide a footing for future research into clinical translation.

## Introduction

Dopamine D_1_ receptor (D_1_R) agonists, in humans and rodents, are known for their role in enhancing cognition (Arnsten et al., 1994; Murphy et al., 1996; Rosell et al., 2015; Arnsten et al., 2017; Yang et al., 2022b; Bransom et al., 2024). These ligands have the potential to activate internal signaling cascades that may be involved in regulating cognitive-related behavior. The D_1_R is a Gα_S/OLF_-coupled GPCR receptor, and its activation results in the production of cAMP (canonical) and β-arrestin recruitment (noncanonical; Mailman et al., 1998; Lefkowitz and Shenoy, 2005; Yang, 2021; Yang et al., 2021; Yang et al., 2022a,b,c). A ligand that disproportionately activates two or more internal signaling cascades after binding to a single receptor is termed to be functionally or signaling biased (Mailman et al., 1998; Urban et al., 2007). Previously, we reported on the effect of two selective D_1_ ligands with different signaling profiles: 2-methyldihydrexidine (2MDHX) and PF-06256142 (PF). 2MDHX is a classical full D_1_ agonist for cAMP synthesis and β-arrestin recruitment (Knoerzer et al., 1995; Yang et al., 2021) whereas PF has relatively high intrinsic activity for cAMP synthesis, but minimal or no activity for β-arrestin recruitment (Davoren et al., 2018; Bransom et al., 2024). The development of functionally selective/biased ligands has expanded our capacity to discover drug candidates with improved therapeutic indices and isolate the functional impact of downstream signaling pathways. Our understanding of how biased signaling impacts components of cognition across behavioral tasks remains, however, unclear.

We have attempted to address this question by testing several D_1_ agonist compounds on related behavioral tasks that measure simple cognitive performance. This included a spatial working memory (WM)-related delayed alternation response paradigm in the T-maze (Yang et al., 2021; Yang et al., 2022b; Cimino et al., 2023) and a temporal order memory-related novel object recognition task in the open field chamber (Bransom et al., 2024). In both tasks, D_1_ agonists recovered poor performance, but it is unclear if these desirable effects extend to more complex tasks that require the integration of cognitive processes. The current study was designed to use the touchscreen-based Trial-Unique Nonmatching-to-Location (TUNL) task to test the hypothesis that a highly biased ligand (PF) would have differential effects from a compound that was not highly functionally selective (2MDHX). This behavioral paradigm requires subjects to maintain the location of the stimuli exposed during the sample phase and recall this information during the choice phase following a delay (Talpos et al., 2010; Oomen et al., 2013; Dexter et al., 2022). Each trial has a unique combination of conditions that are counterbalanced throughout the session to include a variety of trials that vary in difficulty and order. The complexity and the cognitive demands of this task are significantly greater, providing an ideal context for testing compound effects on integrated cognitive processes.

Our specific questions were as follows: (1) does either and or both compounds improve task performance, (2) do these D_1_ agonists change goal-directed response duration, and (3) investigate if 2MDHX or PF differentially impact task engagement. Our results indicate distinct compound- and dose-induced differences in task performance, latency, and engagement, offering valuable insights to guide the optimization of therapeutic regimens and inform future targeted drug development strategies.

## Materials and Methods

### Subjects

A total of 13 male Fischer 344 rats (NIA) were used. They were 157 to 436 d old at the start of the experiments and their weight on the first day of vehicle testing ranged between 318 and 367 g. This sample size was predetermined based on previous studies (Yang et al., 2021; Yang et al., 2022a; Cimino et al., 2023; Bransom et al., 2024). Of these subjects, 10 completed all compound testing experiments. The remaining three subjects were killed at a humane endpoint due to health issues unrelated to the study. Although they did not complete all compound testing experiments, their available data points were included in the final analyses. Subjects were provided with a limited diet of Bio-Serv rat chow to maintain their body weight at 80% of free feeding body weight (4 g of food per 100 g total body weight) following completion of their behavioral session. This was conducted to permit food to be used as motivation but was given 100% of free feeding body weight during rest days. Highly palatable rewards (chocolate flavor sucrose, Bio-Serv) used during the test phase were introduced prior to behavioral training. All animal care and experimental procedures followed the National Institutes of Health Guide for the Care and Use of Laboratory Animals, The Pennsylvania State University College of Medicine Animal Resources Program, and were reviewed and approved by IACUC of The Pennsylvania State University College of Medicine. Subjects were gentled and habituated to laboratory conditions prior to the start of experimental proceedings, housed individually, maintained on a 12 h light/dark cycle, and provided with water *ad libitum*.

### Compound preparation and administration

Compounds were prepared daily to maintain compound integrity and prevent oxidation. 2-Methyldihydrexidine (2MDHX), an analog of dihydrexidine, was synthesized by modifications of published procedures (Knoerzer et al., 1995; Yang et al., 2021). PF-06256142 (PF) is one in a series of noncatechol D_1_ agonists discovered by Pfizer (Davoren et al., 2018) and was obtained through an MTA. Compounds were prepared as stock solutions of 100 mM in DMSO and stored at −80°C in the dark. For use, they were diluted in 0.1% ascorbic acid vehicle using a log-graded dose range (1, 10, 100, 1,000, and 10,000 nmol/kg) suggested by prior studies (Yang et al., 2021; Yang et al., 2022a; Cimino et al., 2023; Bransom et al., 2024) and administered subcutaneously to minimize the role of oral bioavailability and first pass metabolism.

### TUNL task

#### Apparatus

Two easy-install systems for touch screen systems (Model 80604A-20-41) were assembled side-by-side according to manufacturer instructions. Within each of the eight independently functioning chambers, second-generation Bussey–Saksida automated touchscreen operant chamber for rats (Model 80604A) was installed, programed, and tested to ensure proper functionality (Talpos et al., 2010; Oomen et al., 2013; Dexter et al., 2022). The touchscreen chamber was used to facilitate and collect data during TUNL task pretraining and staged sessions. Chambers and waste trays were cleaned immediately following each subject's testing session using 70% EtOH. Additionally, the apparatus was inspected monthly, updated, and serviced as needed to maintain and ensure proper data collection.

#### Pretraining

In preparation for participation in experimental proceedings, subjects began a series of pretraining sessions to increase subject motivation, habituate them to the touchscreen chamber, and become familiar with initiating TUNL task trials. First, we increased subject motivation by enrolling subjects in a restricted diet in which they were given Bio-Serv rat chow portions calculated as 80% of daily baseline weight. Subjects were handled and allowed to acclimate to the reduced diet for three consecutive days. Next, rats were individually placed in their own chamber for 30 min with all internal lights turned off and with the reward tray filled with chocolate-flavored sucrose pellets. This chamber habituation phase occurred across 2–3 exposure sessions. To learn how to initiate and participate in TUNL task, subjects participated in ∼3 d of initial touch training in which they were trained to nose poke the touchscreen in response to the screen lighting up. These hour-long sessions were rewarded with the same chocolate-flavored pellets as the previous sessions and included an intertrial interval (ITI) of 20 s between trials.

After successful completion of subject habituation to the chamber and learning how to nose poke the touchscreen, subjects started a series of training sessions that required precise initiation of predetermined locations on the screen. During these must touch sessions, and for the remainder of the study, a 5 × 3 panel was inserted on top of the screen to create a grid of windows that the subjects will interact with for each 1 h session. Must touch training was broken down into three stages: must touch 1 × 1, must touch 4 squares, and must touch 5 × 3. In must touch 1 × 1, all windows were lit, and subjects were required to nose poke a single illuminated box within the 5 × 3 grid. Next, in must touch 4 squares, the rats must complete stage 2 by nose poking a single illuminated box within the 2 × 2 square window. Finally, in stage 3 of must touch training, a single box was randomly illuminated on the screen while all the other windows remained dark. Subjects were rewarded after correctly selecting the lit box and experienced an ITI between trials. This process continued until the 1 h session was complete.

Following completion of the must touch trial series, rats began the must initiate portion of learning. To establish an association between treat delivery, chamber lights, touching the stimulus, and sound pertaining to the full version of the trial, food was delivered with the tray light on. Subjects must poke the illuminated stimulus to receive a reward and exit the food tray following consumption to initiate the next trial. Finally, the last pretraining session consisted of punishing incorrect selection of stimuli. If the rats selected a nonilluminated box, the chamber light turned on and a 5 s time out period ensued. Failed trials initiated a correction trial in which the rat underwent the same trial they previously failed, paralleling the full version of the task.

#### Staged TUNL task training

The second half of TUNL task training consists of exposing subjects to various levels of trial difficulty, including changes in stimuli separation distance, pattern configuration, and time delay. Subjects were required to reach a performance threshold, which varies slightly by the training phase type, to advance forward in the training paradigm. Generally, a performance threshold of ≥60% was selected to ensure that the subject was performing greater than chance during the vehicle sessions which demonstrated understanding of the task itself. Therefore, during compound testing sessions, large changes in session accuracy can be attributed to effects induced by the dose and compound administered rather than the difficulty associated with the task itself. The remaining stages were consistent with the preceding setup which included sound and light exposure upon entrance to the chamber, following nose poke of touchscreen, and prior to reward for the entirety of the 1 h session duration.

Immediately following the last pretraining session, subjects began training based on separation distance between the sample and choice stimuli. Large separation sessions consisted of trials that only contained horizontal separation distances of three windows between the sample and choice stimuli. Following a nose poke to the illuminated sample stimuli, the stimuli was turned off, and the rat was required to nose poke the lit food tray to initiate the variable delay. A 0.5 s delay occurred during this training stage and was followed by appearance of both the sample and choice stimuli. If a rat nose poked the correct choice stimuli (i.e., the novel illuminated box), a reward was dispensed into the food tray. Once the reward was claimed, a 5 s ITI occurred before the start of the next trial. Conversely, if the rat poked the incorrect choice stimuli (i.e., the original exposure stimuli), the house lights switched on, a 5 s time out period was initiated, and the subject was required to repeat the failed trial configuration in a correction trial. To pass this stage, subjects were required to have an overall correct score of 50% or higher for two consecutive days. The same premise was repeated for the medium separation training session which included a two-window separation between sample and test stimuli. Subjects needed an overall performance score of 60% or higher for 2 consecutive days to move on to the next training block.

**Figure 1.**
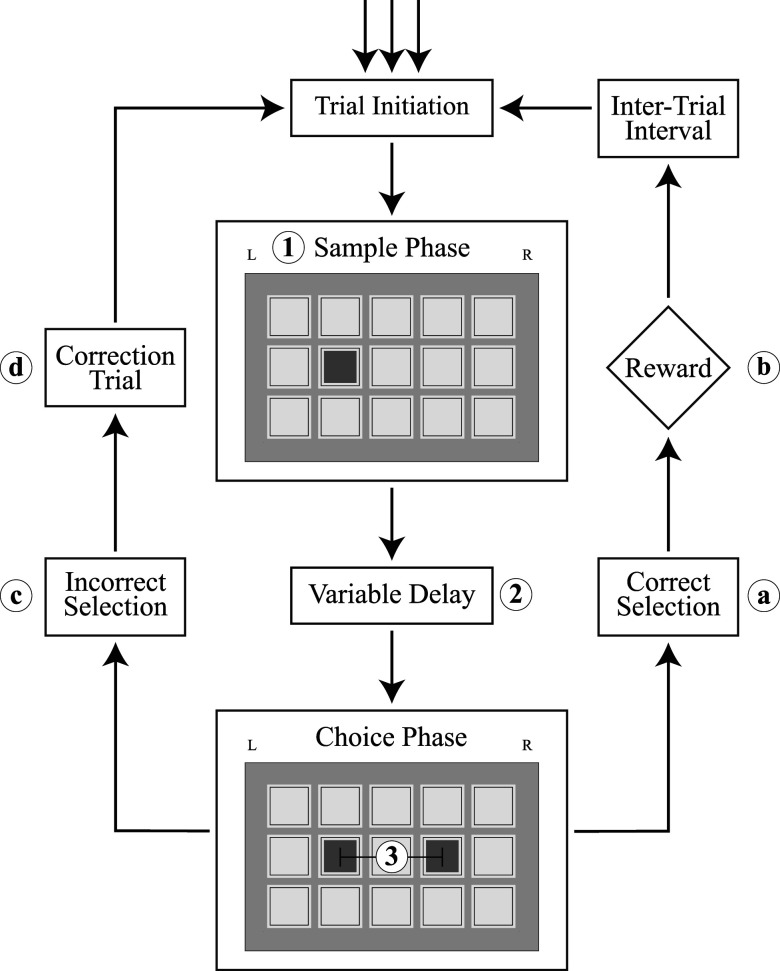
TUNL task trial schematic. Trial factors (**1, 2, 3**) are manipulated for each unique trial condition and influence the difficulty of each trial. These factors are randomly selected and counterbalanced throughout the session for location/side of the initial sample stimuli (**1**), variable delay (**2**) between the sample and choice phase, and separation distance (**3**) between stimuli in the choice phase. Trial-based behavioral parameters collected and assessed (**a, b, c**) include correct (**a**) and incorrect (**c**) choice response latency and reward collection latency (**b**). Note that time events are characterized into standard and correction trials for behavioral parameter assessment.

The remaining training sessions prepared the subjects for trial-dependent separation distance and delay time combinations that influence trial difficulty. During various separation training, the rats completed as many trials as possible within the 1 h timeframe that contained trials of different separation distances and a delay period of 0.5 s. Trials over the course of the session were balanced in initial sample location and separation pattern quantity. Upon 2 consecutive days achieving performance of 60% or higher, subjects started a new iteration of various separation training that contained a longer delay period of 5.0 s. Once again, to pass this training block, subjects were required to achieve a score of 60% or higher for 2 consecutive days. Finally, the last training block contained the full version of the TUNL task paradigm utilized during compound testing. These sessions, and all future trial sessions, contained both delay times of 0.5 and 5.0 s and pattern separation distances of 0, 1, and 2, as shown in Figure 1. To complete the final round of training, subjects must achieve a score of 60% or higher for 2 consecutive days. Trials that contain shorter pattern separation distances and longer delay times create more challenging trials due to an increased demand in pattern discrimination ability and memory maintenance. In contrast, trials that contain larger pattern separation distances and shorter delay times create easier trials and do not require the same level of cognitive effort to successfully complete the trial. Trial separation distance and delay, order, and frequency of trial pairings are randomized and counterbalanced throughout each session to ensure that the session is relatively balanced in terms of trial difficulty. By creating these predefined competency thresholds, subjects are held to the same level of proficiency at each stage. As a result, this demonstrates that subjects are performing the task higher than mere chance.

### Experimental design

Rats were trained once daily and allowed to rest over the weekend. Subjects were acclimated to all procedures and experimental paradigms. First, mock sessions were conducted to habituate subjects to injection discomfort via a subcutaneous needle prick and then prompted to complete a TUNL task session. Contingent upon meeting ≥60% overall correct threshold, subjects advanced to a vehicle administration and TUNL task session on the following day. If performance during the vehicle session was ≥60%, the drug injection and TUNL session occurred the next day. Subjects during mock or vehicle sessions that did not perform better than 60% repeated these sessions until they were able to meet the arbitrary threshold. All subject sessions were started within 10 min of mock, vehicle, or drug administration. After a drug test session was completed, subjects underwent a 5 d minimum “washout” period prior to retesting that animal with a different compound and dose combination. During this washout period, rats continued their daily behavioral training without any compound or vehicle administration.

Compound condition and dose combinations examined using the TUNL task for each subject followed a factorial mixed model approach. Each subject received a mock, vehicle, and drug session for both compounds and dose combinations. Subjects were randomly assigned either 2MDHX or PF as their initial group in order to balance first-exposure effects to either compound. In addition, the number of subjects within these groups was counterbalanced. Subjects received both compounds at the same dose prior to moving on to a new dose. The first dose tested was 10 nmol/kg due to prior results indicating that this dose is most likely to improve cognition (Yang et al., 2021; Yang et al., 2022a; Cimino et al., 2023; Bransom et al., 2024). The remaining four doses (1, 100, 1,000, and 10,000 nmol/kg) were then randomly ordered for each subject. Compound assignment order from the start of the study remained consistent for the remainder of the study and determined which compound condition is always tested first. A washout period of at least 5 d occurred following administration of each unique compound and dose combination.

### Data analysis

Analysis of TUNL task data was conducted following classification of measured parameters in a session-by-session and trial-by-trial manner. To isolate drug-related changes in task performance, data was separated into session and trial-based analyses because session level accuracy values only provide partial information regarding session difficulty conditions. Session parameters collected represent an average value for the variables recorded with respect to time (1 session, 60 min; 3 blocks, 20 min each). Since this task requires sustained cognitive effort to remain engaged in the task, we divided engagement parameters into three arbitrary 20 min time blocks to investigate if task participation remained consistent throughout the 1 h session for summary statistics. In addition, we also analyzed our data in a trial-by-trial manner. This analysis considers a subjects current and previous trial difficulty parameters effect on individual trial performance because individual trial difficulty may impact subsequent trial performance. Individual factors included in each statistical test and model are elaborated further in the following paragraphs. All statistical analysis was performed and modeled using IBM SPSS Statistics (version 30.0), GraphPad Prism (version 10.4.2), and Adobe Illustrator (2025). Subjects’ vehicle sessions, preceding their respective compound sessions, were merged to reduce baseline variability. The figures and tables report mean ± standard error of the mean (SEM) which was calculated from raw data unless otherwise specified. Model-estimated pairwise contrasts and SEs are reported and presented on the link (linear predictor) scale, along with the corresponding *p* value. Predicted correct trial probabilities are reported and presented on the original scale. All statistical tests conducted used a 95% confidence interval. A *p* value of *p* < 0.05 was classified as statistically significant, and least squared difference multiple comparisons were adjusted for using Bonferroni’s correction.

#### Based on session

The analysis of session data targeted differences in session engagement parameters and overall performance accuracy, i.e., the correct choice rate over the course of a session. Session engagement parameters include the number of trials attempted which can be further divided into two summary variables: the number of novel trials initiated and the number of correction trials attempted. Since these count variables do not follow a normal distribution, we used generalized linear mixed effects models (GLMMs) with a Poisson distribution and log link to investigate compound- and dose-induced differences in engagement parameters between compounds at the same dose and between each unique combination to vehicle. This was conducted at the session and block level and contained fixed effects of unique compound and dose combination. Random effects of subject and session group were used to account for subject and time-based variability. Session group is an arbitrary value linking a subject’s compound and preceding vehicle session. Additionally, because dopamine is heavily involved in the regulation of motor movement, we measured the total number of beam breaks in the testing chamber to quantify subject movement. The total number of beam breaks refers to the sum of front and back infrared beam breaks with the front beam sensor closest to the touchscreen and the back beam sensor closest to the feeder. We used a Poisson distribution with a log link and the same fixed and random effects as the previous analysis to investigate differences in the total number of beam breaks between each unique drug and dose combination. In addition, we also used the same fixed and random effects to investigate the effect of each unique compound and dose combination on session accuracy. Since this data fits an approximate normal distribution, we used a GLMM with a normal/Gaussian distribution and identity link.

#### Based on trial

Trial-based data analysis was conducted to incorporate subjects previous and current trial difficulty parameters into our interpretation of compound and dose effects. Trial difficulty parameters included trial sample side (left, right), trial delay (0.5 s, 5.0 s), separation distance (1, √2, 2, √5, 3, √10, √13), the probability of a subject making the correct choice from sample location (0.500, 0.571, 0.714, 0.786, 1), and time block (1, 2, 3). Separation distance is quantified as the straight-line difference between the sample and choice stimuli coordinates known as Euclidean distance. Separation distance for each trial was calculated as *d* = √[(*x*_2_ − *x*_1_)^2^ + (*y*_2_ − *y*_1_)^2^], where (*x*_1_, *y*_1_) is the coordinate of the sample stimuli and (*x*_2_, *y*_2_) is the coordinate of the choice stimuli. This measurement represents both horizontal and vertical distance as a single variable. The probability of a subject making the correct choice based on initial sample location is based on retrospective and prospective WM processes, side bias, and distance between samples (Bennett et al., 2023).

We developed a GLMM with a binomial distribution and logit link to examine the effects of several parameters on the probability of selecting the correct choice stimuli in a compound and dose-dependent manner. This model included fixed effects of unique compound and dose combination, trial sample side, delay, separation distance, delay × separation distance, probability of a subject making the correct guess from sample location, block, previous trial sample side, previous delay, previous separation distance, previous delay × previous separation distance, previous probability of a subject making the correct guess from sample location, and previous choice outcome (0 = incorrect, 1 = correct, 999 = correction trial). Random effects of subject and session group were used to account for subject and time-based variability.

To investigate the compound and dose-dependent effects on response latency to the choice and reward stimuli, we first split correct and incorrect trials into two subcategories: standard trials and correction trials. Following an incorrect selection, subjects are re-exposed to the failed trial and provided with additional opportunities to successfully learn this trial difficulty combination before proceeding to a novel trial. These trials are termed correction trials. Response latency was analyzed separately for standard and correction correct trials, incorrect trials, and reward collection. These time metrics are vital as they may indicate which underlying neural processes are influencing trial success or failure at baseline and as a result of compound administration. We used a gamma distribution with a log link and the same fixed and random effects elaborated on in the previous paragraph (Lo and Andrews, 2015).

## Results

### Effects of 2MDHX and PF on memory-related performance accuracy

**Figure 2.**
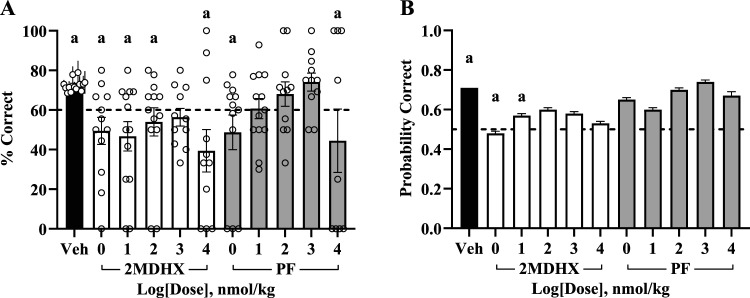
D_1_ agonists change session and trial accuracy. ***A***, Session accuracy. Each bar represents the average (mean ± SEM) number for each parameter with dots representing each individual rat. Individual vehicle data points are the average (mean ± SEM) of all vehicle sessions preceding each subject's respective compound sessions. Note the dashed line at the accuracy threshold (60%). ***B***, Predicted probability of correct trial choice selection. Each bar represents the average (mean ± SEM) predicted probability of selecting the correct choice which is based on model generated estimates in its original scale. Note the dashed line at 0.5 which indicates chance for this behavioral assay. The letter a denotes a significant (*p* < 0.05) difference in performance between vehicle and unique drug and dose combination whereas the letter b denotes a significant (*p* < 0.05) difference between 2MDHX and PF at the same dose.

First, we investigated the effects of 2MDHX and PF on TUNL task accuracy (Fig. 2*A*, Table 1). A higher session accuracy score demonstrates better overall performance than a lower score. The vehicle session preceding compound administration requires an overall session score of 60% or higher to advance, thereby anchoring vehicle session average above mere chance. First, we compared differences in session performance between unique compound and dose combinations against vehicle sessions. Subjects treated with 2MDHX had a significant decrease in session accuracy at the doses of 1 (*β* = −22.98, SE = 6.05, *t*_(225)_ = −3.80, *p* = 0.003), 10 (*β* = −25.70, SE = 5.83, *t*_(225)_ = −4.41, *p* ≤ 0.001), 100 (*β* = −18.44, SE = 5.83, *t*_(225)_ = −3.16, *p* = 0.027), and 10,000 (*β* = −33.02, SE = 6.29, *t*_(225)_ = −5.25, *p* ≤ 0.001) nmol/kg. Subjects treated with PF at 1 (*β* = −23.71, SE = 6.05, *t*_(225)_ = −3.92, *p* = 0.002) and 10,000 (*β* = −27.99, SE = 6.90, *t*_(225)_ = −4.05, *p* = 0.001) nmol/kg performed significantly lower than vehicle. Overall, subjects treated with either compound performed similarly or worse than vehicle treated subjects, and there were no significant differences between compounds at the same dose.

**Table 1.** Effect of D_1_ agonists on overall TUNL session accuracy and estimated correct trial probability

Drug	Dose (nmol/kg)	Overall session accuracy %		Estimated correct trial probability	
		*N*	Mean ± SEM	*N*	Mean ± SEM
Vehicle	0	120	72.4 ± 1.0	1,467	0.71 ± 0.00
2MDHX	1	12	49.5 ± 6.8	132	0.48 ± 0.01
	10	13	46.7 ± 7.4	199	0.57 ± 0.01
	100	13	54.0 ± 7.1	131	0.60 ± 0.01
	1,000	11^(1)^	56.3 ± 4.5	165	0.58 ± 0.01
	10,000	11	39.4 ± 10.7	58	0.53 ± 0.01
PF	1	12	48.7 ± 8.7	117	0.65 ± 0.01
	10	13	60.7 ± 5.1	234	0.60 ± 0.01
	100	11	68.1 ± 6.2	89	0.70 ± 0.01
	1,000	11^(1)^	74.1 ± 4.6	112	0.74 ± 0.01
	10,000	9^(2)^	44.4 ± 16.0	14	0.67 ± 0.02

Overall session accuracy values represent the raw mean ± SEM, whereas trial probability values represent the mean ± SEM of predicted probabilities generated by the transformed model in the original scale. Model for session accuracy: *F*_(10,225)_ = 7.81, *p* ≤ 0.001. Model for estimated correct trial probability: *F*_(53,1628)_ = 1.44, *p* = 0.022; significant fixed effects are as follows: compound dose combination (*F*_(10,1628)_ = 3.21, *p* ≤ 0.001), trial delay (*F*_(1,1628)_ = 3.91, *p* = 0.048), previous trial delay × previous trial separation distance (*F*_(7,1628)_ = 2.19, *p* = 0.033). Each superscript number in parentheses indicates the number of subjects that underwent session testing but did not initiate any trials.

All our GLMMs constructed to evaluate session parameters were unable to include individual trial difficulty in the analysis. Since difficulty varies by trial, and trial composition is not identical between sessions, our trial-by-trial analysis will shed light on what factors contribute to these behavioral differences. Therefore, we incorporated previous and current trial difficulty parameters at a trial-by-trial level and investigated compound and dose differences of predicted probability for selecting the correct choice stimuli (Fig. 2*B*, Table 1). Except for 2MDHX at 1 nmol/kg, subjects had an average predicted probability of performing at or above chance (0.5) for all compound and dose combinations. We found a significant decrease in predicted probability for subjects treated with 2MDHX at 1 (*β* = −0.96, SE = 0.27, *t*_(1628)_ = −3.60, *p* = 0.005) and 10 nmol/kg (*β* = −0.74, SE = 0.24, *t*_(1628)_ = −3.14, *p* = 0.026) compared with vehicle. Although not significant, we observed a general increase in predicted probability for PF-treated subjects in the middle dose range (10−1,000 nmol/kg). Interestingly, the predicted probability of selecting the correct choice was no different between blocks (*F*_(2,1628)_ = 1.41, *p* = 0.244), suggesting that it was not time dependent and remained similar throughout the duration of the session. This contrasts with our engagement parameter and response latency analyses that, as reported below, do demonstrate time-dependent changes throughout the session.

### Characterization of correct, incorrect, and reward response latencies

Following investigation of compound induced changes in overall session accuracy and trial-based probability of selecting the correct choice stimuli, we investigated the impact of compound and dose combinations on trial response latencies. Analyses of subjects’ correct, incorrect, and reward latencies were grouped by standard or correction trial type. Investigation of these response times was conducted independent of one another due to potential differences in underlying biological implications. For correct standard trials, we interpreted shorter response latency as a marker of high discrimination capability (between sample and choice stimuli) whereas long response latency demonstrated a decreased discrimination ability with subjects requiring additional time to select the correct answer. For incorrect standard trials, we interpreted shorter response latency as a failure to discriminate between stimuli, which may be due to trial difficulty, while longer duration may represent more time spent contemplating their choice. Interpretation of response duration for correct and incorrect correction trials adds additional nuance because subjects were exposed to the current trial conditions in the previous trial. Shorter correct image response latency implies strong retrospective WM whereas longer latency may derive from trial difficulty, decreased motivation, or poor WM. Shorter incorrect response latency implies poor discrimination and retrospective WM whereas longer latency may indicate increased time debating stimuli choice or poor attention to the task. Finally, for standard and correction trial reward collection latency, we interpreted shorter reward latency as increased reward seeking behavior and task drive whereas longer collection latency demonstrated decreased.

**Figure 3.**
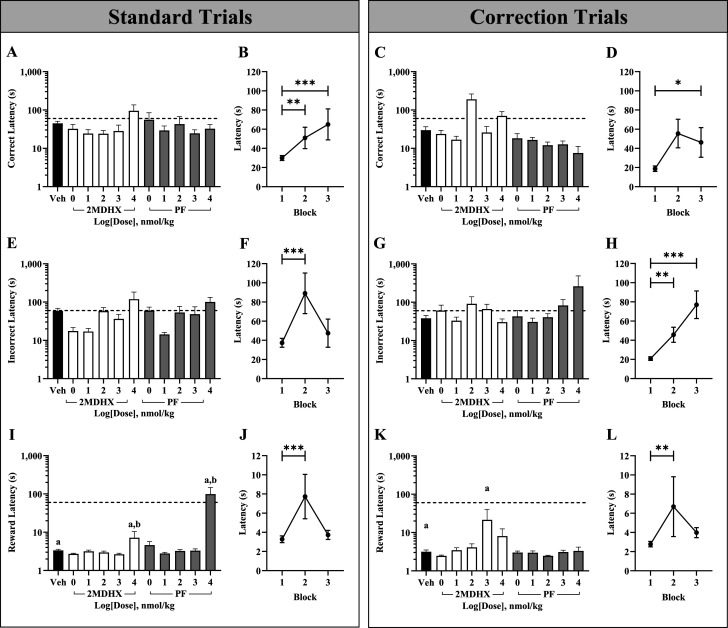
Compound- and dose-induced changes in image and reward response latency. Standard trial correct (***A***), incorrect (***E***), and reward response latency (***I***). Average image response latency by block for standard correct trials (***B***), incorrect trials (***F***), and reward collection (***J***). Correction trial correct (***C***), incorrect (***G***), and reward response latency (***K***). Average image response latency by block for correction correct trials (***D***), incorrect trials (***H***), and reward collection (***L***). In panels ***A***, ***C***, ***E***, ***G***, ***I***, and ***K***, each bar represents the average (mean ± SEM) image or reward response latency. In addition, the dashed line indicates average responses over 60 s. The letter a denotes a significant (*p* < 0.05) difference in performance between vehicle and unique drug and dose combination whereas the letter b denotes a significant (*p* < 0.05) difference between 2MDHX and PF at the same dose. In panels ***B***, ***D***, ***F***, ***H***, ***J***, and ***L***, each point represents the average latency by block ± SEM. Significant differences between blocks are denoted by *p < 0.05, **p < 0.01, ***p < 0.001.

First, we investigated potential differences in standard trial correct image response latency across compound and dose conditions (Fig. 3*A*, Table 2), and we found no significant differences. However, as shown in Figure 3*B*, correct image response latency significantly changes throughout the course of the session (*F*_(2,1069)_ = 16.40, *p *≤ 0.001), such that response latency during block 1 was significantly shorter than blocks 2 (*β* = −0.56, SE = 0.18, *t*_(1069)_ = −3.08, *p* = 0.006) and 3 (*β* = −0.98, SE = 0.18, *t*_(1069)_ = −5.53, *p *≤ 0.001), though we observed no significant difference between blocks 2 and 3 (*β* = −0.42, SE = 0.21, *t*_(1069)_ = −1.98, *p* = 0.145). Block 1 had the shortest average latency (*x̅* = 29.94, SEM = 2.63), with Blocks 2 (*x̅* = 50.94, SEM = 11.14) and 3 (*x̅* = 64.96, SEM = 16.16) steadily increasing.

**Table 2.** Effect of compound and dose combinations on average image response latencies

Compound	Dose (nmol/kg)	Mean standard trial latency (s) ± SEM				Mean correction trial latency (s) ± SEM			
		*N*	Correct	*N*	Incorrect	*N*	Correct	*N*	Incorrect
Vehicle	0	800	45 ± 6	323	58 ± 10	268	30 ± 7	499	38 ± 7
2MDHX	1	40	32 ± 10	37	17 ± 4	29	24 ± 6	75	60 ± 23
	10	56	24 ± 6	43	17 ± 3	35	17 ± 4	120	33 ± 8
	100	54	24 ± 5	35	58 ± 14	26	190 ± 73	60	91 ± 49
	1,000	49	28 ± 12	32	36 ± 11	22	26 ± 11	104	67 ± 22
	10,000	24	95 ± 41	24	119 ± 64	17	70 ± 20	23	30 ± 6
PF	1	50	55 ± 30	31	59 ± 14	23	18 ± 6	59	43 ± 18
	10	78	29 ± 9	47	14 ± 2	39	17 ± 3	127	31 ± 8
	100	56	43 ± 24	25	53 ± 23	19	12 ± 3	22	41 ± 10
	1,000	70	25 ± 6	22	48 ± 27	17	13 ± 3	34	83 ± 34
	10,000	12	32 ± 9	9	101 ± 32	4	8 ± 4	4	261 ± 227

All standard and correction trial response latency averages represent the raw mean ± SEM. Model for standard trial correct image response latency: *F*_(53,1069)_ = 2.10, *p* ≤ 0.001; significant fixed effects are: time block (*F*_(2,1069)_ = 16.40, *p* ≤ 0.001), trial sample side (*F*_(1,1069)_ = 6.91, *p* = 0.009), trial delay (*F*_(1,1069)_ = 10.61, *p* = 0.001), probability of selecting the correct choice for the previous trial (*F*_(4,1069)_ = 3.41, *p* = 0.009). Model for standard trial incorrect image response latency: *F*_(53,505)_ = 1.20, *p* = 0.161; significant fixed effects are: time block (*F*_(2,505)_ = 8.45, *p* ≤ 0.001), trial delay (*F*_(1,505)_ = 9.36, *p* = 0.002). Model for correction trial correct image response latency: *F*_(33,465)_ = 0.96, *p* = 0.529; there are no significant fixed effects. Model for correction trial incorrect image response latency: *F*_(33,1093)_ = 3.32, *p* ≤ 0.001; significant fixed effects are as follows: compound and dose (*F*_(10,1093)_ = 2.52, *p* = 0.005), time block (*F*_(2,1093)_ = 11.66, *p* ≤ 0.001), previous choice score (*F*_(1,1093)_ = 7.50, *p* = 0.006).

Then, we investigated differences in standard trial incorrect image response latency (Fig. 3*E*, Table 2) and found no significant differences between conditions. However, as shown in Figure 3*F*, the standard trial incorrect image response latency was significantly different between blocks (*F*_(2,505)_ = 8.45, *p *≤ 0.001). Between blocks 1 and 2, incorrect image response latency was significantly shorter during block 1 compared with block 2 (*β* = −1.14, SE = 0.28, *t*_(505)_ = −4.09, *p* = <0.001). There was, however, no significant difference in response latency between blocks 1 and 3 (*β* = −0.46, SE = 0.31, *t*_(505)_ = −1.48, *p* = 0.418) or between blocks 2 and 3 (*β* = 0.68, SE = 0.357, *t*_(505)_ = 1.909, *p* = 0.170). Block 1 had the shortest average latency (*x̅* = 37.40, SEM = 4.63), with block 2 (*x̅* = 89.05, SEM = 21.20) increasing in latency before decreasing in block 3 (*x̅* = 47.45, SEM = 14.72). Together, this suggests that subjects’ estimated response latency increases between blocks 1 and 2 and then plateaus between blocks 2 and 3.

Next, we investigated dose- and compound-dependent differences in correct (Fig. 3*C*, Table 2) response latency for correction trials. We found no significant differences in correction trial correct response latency between conditions. However, correct image response latency for correction trials was different between blocks (*F*_(2,465)_ = 2.91, *p* = 0.056), as shown in Figure 3*D*. We observed a significant increase in response latency between blocks 1 and 3 (*β* = −0.79, SE = 0.33, *t*_(465)_ = −2.40, *p* = 0.0499). There was no significant difference in response latency between blocks 1 and 2 (*β* = −0.22, SE = 0.31, *t*_(465)_ = −0.69, *p* = 1.000) or between blocks 2 and 3 (*β* = −0.58, SE = 0.36, *t*_(465)_ = −1.59, *p* = 0.340). Block 1 had the shortest average latency (*x̅* = 18.76, SEM = 2.84), with block 2 increasing in duration (*x̅* = 55.46, SEM = 14.91) before plateauing in block 3 (*x̅* = 46.20, SEM = 15.47).

Then, we investigated compound- and dose-induced differences in incorrect image response latency for correction trials (Fig. 3*G*, Table 2) and observed no differences between conditions. However, once again, the incorrect image response latency for correction trials was significantly different between blocks as shown in Figure 3*H* (*F*_(2,1093)_ = 11.66, *p* ≤ 0.001), with incorrect image response time in block 1 significantly shorter than block 2 (*β* = −0.32, SE = 0.11, *t*_(1093)_ = −2.95, *p* = 0.010) and block and 3 (*β* = −0.52, SE = 0.11, *t*_(1093)_ = −4.77, *p* = <0.001). We observed no significant difference between blocks 2 and 3 (*β* = −0.20, SE = 0.11, *t*_(1093)_ = −1.78, *p* = 0.225). Block 1 had the shortest average latency (*x̅* = 20.85, SEM = 1.85), with blocks 2 (*x̅* = 45.75, SEM = 7.85) and 3 (*x̅* = 76.98, SEM = 14.35) increasing throughout the session.

Finally, in our last set of analyses, we examined compound and dose differences in trial reward collection latency. For standard trials (Fig. 3*I*, Table 3), we found that reward collection latency was significantly longer for subjects treated with 2MDHX at 10,000 nmol/kg compared with vehicle (*β* = 0.74, SE = 0.20, *t*_(1069)_ = 3.72, *p* = 0.003) and for PF at 10,000 nmol/kg compared with vehicle (*β* = 2.40, SE = 0.30, *t*_(1069)_ = 7.89, *p *≤ 0.001). Between compounds of the same dose, subjects treated with 2MDHX at 10,000 nmol/kg took significantly more time for reward collection compared with PF (*β* = −1.66, SE = 0.36, *t*_(1069)_ = −4.58, *p *≤ 0.001). As shown in Figure 3*J*, standard trial reward collection latency was significantly different between blocks (*F*_(2,1069)_ = 10.16, *p* ≤ 0.001). There was a significant increase in reward collection latency between blocks 1 and 2 (*β* = −0.30, SE = 0.07, *t*_(1069)_ = −4.42, *p* ≤ 0.001), but we observed no difference between blocks 2 and 3 (*β* = 0.16, SE = 0.08, *t*_(1069)_ = 1.99, *p* = 0.142) and blocks 1 and 3 (*β* = −0.14, SE = 0.07, *t*_(1069)_ = −2.15, *p* = 0.096). Block 1 had the shortest average collection latency (*x̅* = 3.27, SEM = 0.35) which increased during block 2 (*x̅* = 7.73, SEM = 2.31) and then decreased in block 3 (*x̅* = 3.73, SEM = 0.47).

**Table 3.** Effect of compound and dose combinations on average reward collection latencies

Compound	Dose (nmol/kg)	Standard trial latency (s)		Correction trial latency (s)	
		*N*	Mean ± SEM	*N*	Mean ± SEM
Vehicle	0	800	3.36 ± 0.21	268	3.15 ± 0.34
2MDHX	1	40	2.73 ± 0.10	29	2.45 ± 0.13
	10	56	3.16 ± 0.26	35	3.45 ± 0.57
	100	54	2.95 ± 0.25	26	4.12 ± 0.95
	1,000	49	2.65 ± 0.17	22	21.43 ± 18.55
	10,000	24	7.18 ± 3.21	17	8.05 ± 4.43
PF	1	50	4.62 ± 1.10	23	3.02 ± 0.27
	10	78	2.80 ± 0.19	39	2.98 ± 0.32
	100	56	3.24 ± 0.29	19	2.46 ± 0.09
	1,000	70	3.31 ± 0.37	17	3.09 ± 0.37
	10,000	12	99.35 ± 47.96	4	3.32 ± 0.82

All standard and correction trial reward response latency averages represent the raw mean ± SEM. Model for standard trial: *F*_(53,1069)_ = 3.20, *p* ≤ 0.001; significant fixed effects are as follows: compound and dose combination (*F*_(10,1069)_ = 8.57, *p* ≤ 0.001), time block (*F*_(2,1069)_ = 10.16, *p* ≤ 0.001), previous trial delay (*F*_(1,1069)_ = 7.35, *p* = 0.007). Model for correction trial: *F*_(33,465)_ = 2.43, *p* ≤ 0.001; significant fixed effects are as follows: compound and dose combination (*F*_(10,465)_ = 2.71, *p* = 0.003), time block (*F*_(2,465)_ = 6.20, *p* = 0.002).

**Table 4.** D1 agonist dose-dependent differences in TUNL session engagement parameters

Parameter	Drug	Dose (nmol/kg)	*N*	Mean ± SEM			
				Overall session	Block 1	Block 2	Block 3
Trials attempted	Vehicle	0	120	15.8 ± 0.9	8.7 ± 0.6	3.5 ± 0.3	3.6 ± 0.4
	2MDHX	1	12	15.1 ± 2.3	7.1 ± 1.3	4.4 ± 1.2	3.6 ± 0.9
		10	13	19.5 ± 2.5	8.2 ± 1.4	6.2 ± 1.4	5.2 ± 1.0
		100	13	13.5 ± 3.2	5.8 ± 1.5	4.9 ± 1.5	2.8 ± 0.9
		1,000	11^(1)^	18.8 ± 3.2	10.0 ± 2.1	4.0 ± 0.8	4.8 ± 1.2
		10,000	11	8.0 ± 1.8	4.8 ± 0.2	2.1 ± 0.7	1.1 ± 0.5
	PF	1	12	13.6 ± 1.7	6.3 ± 1.2	4.2 ± 1.4	3.2 ± 1.3
		10	13	22.4 ± 4.9	9.5 ± 2.3	6.2 ± 1.6	6.7 ± 1.8
		100	11	11.1 ± 1.8	7.1 ± 1.4	2.7 ± 0.8	1.3 ± 0.6
		1,000	11^(1)^	13.0 ± 2.3	5.6 ± 1.2	3.2 ± 1.1	4.3 ± 1.4
		10,000	9^(2)^	3.2 ± 0.6	2.1 ±0.8	0.9 ± 0.3	0.2 ± 0.2
Novel trials	Vehicle	0	120	9.4 ± 0.5	5.7 ± 0.4	1.9 ± 0.2	1.7 ± 0.2
	2MDHX	1	12	6.4 ± 1.2	4.1 ± 0.5	1.3 ± 0.6	1.0 ± 0.6
		10	13	7.6 ± 1.4	4.2 ± 1.0	1.7 ± 0.8	1.8 ± 0.6
		100	13	6.9 ± 1.4	3.2 ± 0.8	2.6 ± 1.0	1.9 ± 0.5
		1,000	11^(1)^	7.4 ± 1.6	5.3 ± 1.0	0.8 ± 0.3	1.3 ± 0.8
		10,000	11	4.4 ± 1.1	2.6 ± 0.6	1.1 ± 0.3	0.7 ± 0.3
	PF	1	12	6.8 ± 1.1	3.7 ± 1.0	2.1 ± 0.9	1.0 ± 0.7
		10	13	9.6 ± 1.8	5.2 ± 1.4	1.9 ± 0.8	2.5 ± 0.7
		100	11	7.4 ± 1.2	5.0 ± 1.1	1.2 ± 0.5	1.2 ± 0.6
		1,000	11^(1)^	8.4 ± 1.6	4.3 ± 0.9	1.8 ± 0.8	2.3 ± 0.9
		10,000	9^(2)^	2.3 ± 0.5	1.8 ± 0.6	0.4 ± 0.2	0.1 ± 0.1
Correction trials	Vehicle	0	120	6.4 ± 0.6	2.9 ± 0.3	1.6 ± 0.2	1.9 ± 0.3
	2MDHX	1	12	8.7 ± 1.5	3.0 ± 0.9	3.1 ± 0.9	2.6 ± 0.6
		10	13	11.9 ± 2.2	4.0 ± 0.8	4.5 ± 1.1	3.5 ± 0.7
		100	13	6.6 ± 1.9	2.6 ± 1.1	2.3 ± 0.9	1.7 ± 0.6
		1,000	11^(1)^	11.5 ± 2.1	4.7 ± 1.3	3.2 ± 0.7	3.6 ± 0.7
		10,000	11	3.6 ± 1.1	2.3 ± 0.7	1.0 ± 0.4	0.4 ± 0.2
	PF	1	12	6.8 ± 1.3	2.6 ± 0.5	2.1 ± 0.9	2.2 ± 1.1
		10	13	12.8 ± 4.5	4.4 ± 1.7	4.2 ± 1.4	4.2 ± 1.6
		100	11	3.7 ± 0.9	2.1 ± 0.5	1.6 ± 0.6	0.1 ± 0.1
		1,000	11^(1)^	4.6 ± 1.3	1.3 ± 0.5	1.4 ± 0.5	2.0 ± 0.7
		10,000	9^(2)^	0.9 ± 0.3	0.3 ± 0.2	0.4 ± 0.2	0.1 ± 0.1

All session and block engagement parameter averages represent the raw mean ± SEM. Each superscript number in parentheses indicates the number of subjects that underwent session testing but did not initiate any trials.

Then, we investigated differences in correction trial reward collection latency (Fig. 3*K*, Table 3). Reward collection latency was significantly longer for subjects treated with 2MHDX at 1,000 nmol/kg compared with vehicle (*β* = 0.96, SE = 0.23, *t*_(465)_ = 4.26, *p* ≤ 0.001). As shown in Figure 3*L*, there was a significant difference in correction trial reward collection latency between blocks (*F*_(2,465)_ = 6.20, *p* = 0.002). Correction trial reward collection latency significantly increased between blocks 1 and 2 (*β* = −0.37, SE = 0.11, *t*_(465)_ = −3.32, *p* = 0.003) but not between blocks 2 and 3 (*β* = 0.10, SE = 0.13, *t*_(465)_ = 0.78, *p* = 1.000) and 1 and 3 (*β* = −0.27, SE = 0.12, *t*_(465)_ = −2.32, *p* = 0.062). Average correction trial collection latency for blocks 1 (*x̅* = 2.78, SEM = 0.29), 2 (*x̅* = 6.69, SEM = 3.12), and 3 (*x̅* = 3.98, SEM = 0.53) followed the same time-based trend as standard trial collection latency.

### 2MDHX and PF induced changes in task engagement

**Figure 4.**
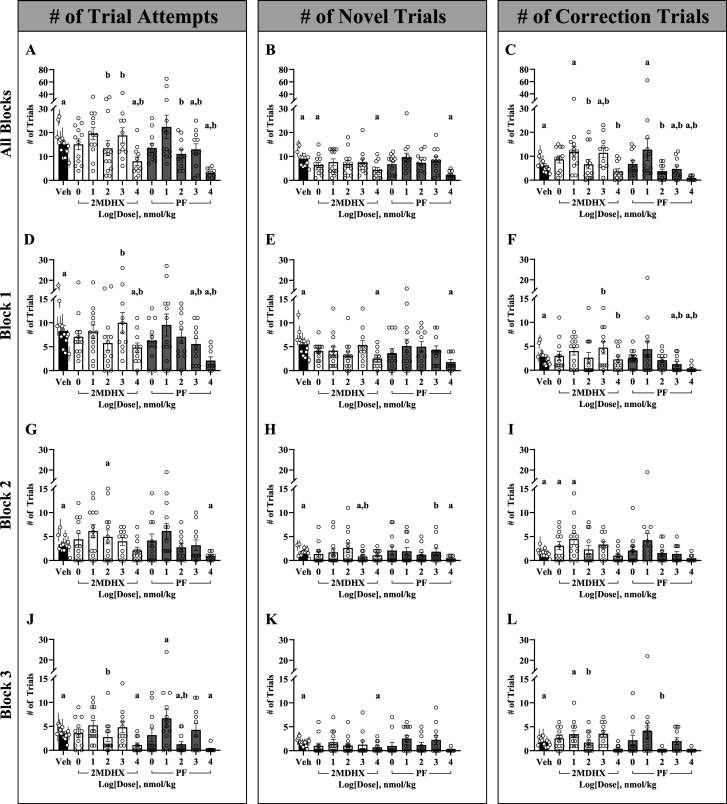
Compound- and dose-dependent changes in session and block task engagement parameters. Number of trials attempted (all blocks, ***A***; block 1, ***D***; block 2, ***G***; and block 3, ***J***), number of novel trials attempted (all blocks, ***B***; block 1, ***E***; block 2, ***H***; and block 3, ***K***), and the number of correction trials attempted (all blocks, ***C***; block 1, ***F***; block 2, ***I***; block 3, ***L***) for the TUNL session. Each bar represents the average (mean ± SEM) number for each parameter with dots representing each individual rat. Vehicle data points for individual subjects are the average (mean ± SEM) of all vehicle sessions preceding their respective compound sessions. The letter a denotes a significant (*p* < 0.05) difference in performance between vehicle and unique drug and dose combination whereas the letter b denotes a significant (*p* < 0.05) difference between 2MDHX and PF at the same dose.

In the final set of analyses, we characterized subject session engagement. The total quantity of trials initiated across each session are variable within the same subject and between subjects. To illuminate the underlying behavioral principles driving differences in number of trial attempts, the number of trials attempted was divided into the number of novel trials initiated and number of correction trials. Then, to determine if our compound and dose combinations changed session engagement, we used a GLMM to compare the number of trials attempted (Fig. 4*A*,*D*,*G*,*J*; Table 4), the number of unique trials initiated (Fig. 4*B*,*E*,*H*,*K*; Table 4), and the number of correction trials attempted (Fig. 4*C*,*F*,*I*,*L*; Table 4) in a condition-dependent manner. In addition, we incorporated performance accuracy, reward response latency, and task engagement results in one table to display overarching trends (Table 5). In general, we interpreted a high number of trials attempted as an indicator of increased task engagement. Conversely, a low number of trials attempted may imply that the compound and dose combination affected the subject’s engagement, that the subject had reduced motivation after several successful trials and stopped initiating more, or that subjects were unable to pass the trial and quit for the remainder of the session. We interpreted a higher number of novel trials initiated as representing high motivation and demonstrates that subjects’ previous trials have been successful since they must pass the previous trial to advance forward in the task. In contrast, a subject that has attempted a moderate number of trials but has a low number of novel trials may indicate that the subject is unable to make the correct choice or has low motivation and as a result, more trial attempts are spent doing correction trials. For our last engagement parameter, a high quantity of correction trials may signal that a subject is persistent in continuing the task regardless of trial accuracy whereas a low number of correction trials indicates better performance upon initial attempt. Finally, we also analyzed the quantity of total beam breaks between unique drug and dose conditions to quantify movement of the subject.

**Table 5.** Summary of performance accuracy, reward response latency, and task engagement results compared with vehicle performance

Drug	Dose (nmol/kg)	% Correct	# of trial attempts	Novel trials		Correction trials	
				# of attempts	Reward response latency	# of attempts	Reward response latency
Vehicle	0	↔	↔	↔	↔	↔	↔
2MDHX	1	**↓**	↔	**↓**	↓	↑	↓
	10	**↓**	↑	↓	↔	**↑**	↔
	100	**↓**	↔	↓	↔	↔	↔
	1,000	↓	↑	↓	↓	**↑**	**↑**
	10,000	**↓**	**↓**	**↓**	**↑**	↓	↑
PF	1	**↓**	↔	↓	↑	↔	↔
	10	↓	↑	↔	↓	**↑**	↔
	100	↔	↓	↓	↔	↓	↓
	1,000	↑	**↓**	↔	↔	**↓**	↔
	10,000	**↓**	**↓**	**↓**	**↑**	**↓**	↔

↔ Indicates baseline or that the unique drug and dose combination compared with vehicle is similar in performance for that session parameter.

**↑** Drug and dose combination significantly increased performance relative to vehicle.

**↓** Drug and dose combination significantly decreased performance relative to vehicle.

↑ Nonsignificant trends toward increased performance relative to vehicle.

↓ Nonsignificant trends toward decreased performance relative to vehicle.

First, we investigated session differences in compound and dose combinations for the number of trials attempted (*F*_(10,225)_ = 12.90, *p *≤ 0.001). Subjects treated with 2MDHX at 10,000 (*β* = −0.66, SE = 0.13, *t*_(225)_ = −5.22, *p* = <0.001) and PF at 1,000 (*β* = −0.33, SE = 0.11, *t*_(225)_ = −3.14, *p* = 0.002) and 10,000 (*β* = −1.71, SE = 0.20, *t*_(225)_ = −8.62, *p *≤ 0.001) nmol/kg attempted significantly less trials than vehicle. When comparing between PF and 2MDHX at the same dose, 2MDHX-treated subjects attempted significantly more trials at 100 (*β* = 0.46, SE = 0.15, *t*_(225)_ = 3.02, *p* = 0.042), 1,000 (*β* = 0.43, SE = 0.14, *t*_(225)_ = 3.01, *p* = 0.044), and 10,000 (*β* = 1.05, SE = 0.24, *t*_(225)_ = 4.47, *p *≤ 0.001) nmol/kg. To incorporate time into all session engagement analyses and uncover potential time-related changes in compound performance, we quantified the number of trials attempted per session and grouped them into three 20 min blocks (Fig. 4*D*,*G*,*J*). In block 1 (*F*_(10,225)_ = 8.69, *p *≤ 0.001), compared with vehicle, subjects performed significantly less trials when administered 2MHDX at 10,000 (*β* = −0.50, SE = 0.17, *t*_(225)_ = −2.99, *p* = 0.047) and PF at 1,000 (*β* = −0.79, SE = 0.15, *t*_(225)_ = −5.23, *p *≤ 0.001) and 10,000 (*β* = −1.59, SE = 0.25, *t*_(225)_ = −6.44, *p *≤ 0.001) nmol/kg, paralleling the overall session results. When comparing between compounds, 2MDHX-treated subjects attempted significantly more trials at 1,000 (*β* = 0.99, SE = 0.20, *t*_(225)_ = 4.94, *p *≤ 0.001) and 10,000 (*β* = 1.10, SE = 0.30, *t*_(225)_ = 3.69, *p* = 0.004) nmol/kg than PF-treated subjects. In block 2 (*F*_(10,225)_ = 4.79, *p *≤ 0.001), we observed an increase in the number of trial attempts for 2MDHX at 100 nmol/kg (*β* = 0.70, SE = 0.19, *t*_(225)_ = 3.69, *p* = 0.004) and a decrease for PF at 10,000 nmol/kg (*β* = −1.53, SE = 0.38, *t*_(225)_ = −4.01, *p* = 0.001) at 10,000 nmol/kg compared with vehicle. We observed no differences in the number of trials attempted in block 2 between PF and 2MDHX. In block 3 (*F*_(10,225)_ = 6.97, *p *≤ 0.001), subjects treated with 2MDHX at 10,000 nmol/kg (*β* = −1.44, SE = 0.31, *t*_(225)_ = −4.57, *p *≤ 0.001) and PF at 100 (*β* = −1.11, SE = 0.30, *t*_(225)_ = −3.70, *p* = 0.004) and 10,000 (*β* = −2.73, SE = 0.72, *t*_(225)_ = −3.77, *p* = 0.003) nmol/kg attempted significantly less trials than vehicle. In contrast, subjects treated with PF at 10 nmol/kg attempted significantly more trials than vehicle in block 3 (*β* = 0.53, SE = 0.16, *t*_(225)_ = 3.27, *p* = 0.018). Between compounds at the same dose, subjects treated with 2MDHX at 100 nmol/kg attempted significantly more trials than subjects treated with PF (*β* = 1.22, SE = 0.37, *t*_(225)_ = 3.31, *p* = 0.016). Generally, we observed a decrease in the overall number of trials attempted for the highest compound doses compared with vehicle with PF-treated subjects attempting less trials than 2MDHX at the highest dose.

Next, we investigated if our compound and dose combinations affected the number of novel trials initiated at the session and block levels. At the session level (*F*_(10,225)_ = 9.41, *p *≤ 0.001), subjects initiated significantly fewer novel trials compared with vehicle when administered 2MDHX at 1 (*β* = −0.43, SE = 0.13, *t*_(225)_ = −3.20, *p* = 0.023) and 10,000 (*β* = −0.82, SE = 0.16, *t*_(225)_ = −5.06, *p *≤ 0.001) and PF at 10,000 (*β* = −1.59, SE = 0.23, *t*_(225)_ = −6.84, *p *≤ 0.001) nmol/kg. We observed no differences in the number of novel trials initiated between compounds at the same dose. In block 1 (*F*_(10,225)_ = 6.36, *p *≤ 0.001), subjects treated with 2MDHX (*β* = −0.77, SE = 0.21, *t*_(225)_ = −3.65, *p* = 0.005) and PF (*β* = −1.39, SE = 0.27, *t*_(225)_ = −5.16, *p *≤ 0.001) at 10,000 nmol/kg performed significantly fewer novel trials compared with vehicle. In block 2 (*F*_(10,225)_ = 4.06, *p *≤ 0.001), subjects initiated significantly less trials following administration of 2MDHX at 1,000 (*β* = −1.35, SE = 0.36, *t*_(225)_ = −3.72, *p* = 0.004) and PF at 10,000 (*β* = −1.72, SE = 0.53, *t*_(225)_ = −3.24, *p* = 0.021) nmol/kg compared with vehicle. Interestingly, in block 2, we found a significant difference in the number of novel trials initiated between 2MDHX and PF that was not captured at the overall session level. At 1,000 nmol/kg (*β* = −1.81, SE = 0.47, *t*_(225)_ = −3.85, *p* = 0.002), 2MDHX subjects initiated significantly less novel trials than PF. Finally, in block 3 (*F*_(10,225)_ = 3.23, *p *≤ 0.001), subjects treated with 2MDHX at 10,000 nmol/kg initiated significantly fewer trials than vehicle (*β* = −1.26, SE = 0.39, *t*_(225)_ = −3.23, *p* = 0.021). Overall, subjects performed fewer novel trials for the outer dose ranges.

The last session engagement parameter we investigated was the number of correction trials attempted. In the overall session analysis (*F*_(10,225)_ = 9.93, *p *≤ 0.001), we compared each unique compound and dose condition to vehicle and between one another at the same dose. 2MDHX at 10 (*β* = 0.46, SE = 0.12, *t*_(225)_ = 3.82, *p* = 0.003) and 1,000 (*β* = 0.50, SE = 0.14, *t*_(225)_ = 3.67, *p* = 0.005) and PF at 10 (*β* = 0.35, SE = 0.11, *t*_(225)_ = 3.06, *p* = 0.037) nmol/kg attempted significantly more correction trials than vehicle sessions. In contrast, subjects treated with PF at 1,000 (*β* = −0.56, SE = 0.17, *t*_(225)_ = −3.27, *p* = 0.018) and 10,000 (*β* = −1.92, SE = 0.37, *t*_(225)_ = −5.16, *p* ≤ 0.001) nmol/kg significantly decreased the number of correction trials attempted. Between 2MDHX and PF at the same dose, 2MDHX subjects attempted significantly more correction trials at doses of 100 (*β* = 0.85, SE = 0.24, *t*_(225)_ = 3.50, *p* = 0.008), 1,000 (*β* = 1.05, SE = 0.22, *t*_(225)_ = 4.87, *p *≤ 0.001), and 10,000 (*β* = 1.51, SE = 0.42, *t*_(225)_ = 3.60, *p* = 0.006) nmol/kg. In block 1 (*F*_(10,225)_ = 4.53, *p *≤ 0.001), subjects treated with PF at 1,000 (*β* = −1.29, SE = 0.30, *t*_(225)_ = −4.37, *p *≤ 0.001) and 10,000 (*β* = −2.19, SE = 0.60, *t*_(225)_ = −3.64, *p* = 0.005) nmol/kg attempted significantly fewer correction trials than vehicle subjects. 2MDHX-treated subjects, at the same doses of 1,000 (*β* = 1.75, SE = 0.36, *t*_(225)_ = 4.90, *p *≤ 0.001) and 10,000 (*β* = 2.07, SE = 0.65, *t*_(225)_ = 3.18, *p* = 0.025) nmol/kg, attempted significantly more correction trials than their analogous PF dose. In block 2 (*F*_(10,225)_ = 4.63, *p *≤ 0.001), 2MDHX-treated subjects at 1 (*β* = 0.99, SE = 0.25, *t*_(225)_ = 3.89, *p* = 0.002) and 10 (*β* = 0.79, SE = 0.21, *t*_(225)_ = 3.84, *p* = 0.002) nmol/kg performed significantly more correction trials than their vehicle counterparts. In our last time-based analysis, subjects treated with 2MDHX at 10 nmol/kg in block 3 (*F*_(10,225)_ = 5.10, *p *≤ 0.001) attempted significantly more correction trials compared with vehicle (*β* = 0.71, SE = 0.22, *t*_(225)_ = 3.25, *p* = 0.020). In addition, those treated with 2MDHX at 100 nmol/kg attempted more correction trials than PF at the same dose (*β* = 3.20, SE = 1.05, *t*_(225)_ = 3.05, *p* = 0.038). Overall, we observed a general decrease in the amount of correction trials attempted throughout the course of the session, regardless of drug and dose combination, except for subjects treated with 1 or 10 nmol/kg of 2MDHX or PF, which tended to increase their amount of correction trials at the overall session and block levels.

Finally, we investigated drug and dose differences in the total number of beam breaks per session (*F*_(10,229)_ = 17.18, *p *≤ 0.001; Table 6). Subjects treated with 2MDHX at 1,000 (*β* = 0.22, SE = 0.02, *t*_(229)_ = 9.12, *p *≤ 0.001) and PF at 1,000 (*β* = 0.15, SE = 0.03, *t*_(229)_ = 6.06, *p *≤ 0.001) and 10,000 (*β* = 0.10, SE = 0.03, *t*_(229)_ = 3.98, *p* = 0.001) nmol/kg had significantly more total beam breaks than vehicle. In contrast, subjects treated with PF at 1 nmol/kg (*β* = −0.12, SE = 0.03, *t*_(229)_ = −4.57, *p *≤ 0.001) had significantly less beam breaks than vehicle. There were no significant differences between compounds at the same dose.

**Table 6.** Average number of total beam breaks

Drug	Dose (nmol/kg)	*N*	Mean total beam breaks ± SEM
Vehicle	0	120	254 ± 11
2MDHX	1	12	244 ± 33
	10	13	309 ± 34
	100	13	207 ± 33
	1,000	12	304 ± 42
	10,000	11	270 ± 31
PF	1	12	211 ± 20
	10	13	312 ± 35
	100	11	245 ± 39
	1,000	12	274 ± 33
	10,000	11	301 ± 35

The total number of beam break averages represent the raw mean ± SEM. The total number of beam breaks is the sum of front and back infrared beam breaks in TUNL task chamber.

## Discussion

The primary goal of this study was to investigate the effect of two selective D_1_ agonists with different signaling profiles on a complex behavioral task requiring integrated cognitive processes. To assess our objectives, we used an automated touchscreen behavioral operant chamber to simultaneously test spatial WM and pattern separation processes across a dose range previously shown to enhance cognition (Yang et al., 2021; Yang et al., 2022b; Cimino et al., 2023; Bransom et al., 2024).

One important criticism of rodent-based cognitive research is face validity of the chosen behavioral assessment. How well the behavioral assessment reflects the complex changes in humans is a common and valid concern when seeking clinical translation of research in murine subjects. We employed TUNL task in our assessment of integrated cognitive processes specifically due to its high face validity, steering away from traditional ethologically based behavioral assessments. The utilization of touchscreen-based behavioral paradigms for bench scientists and clinicians alike provides a unique opportunity to use similar assessments across species (Dumont et al., 2021; Lee et al., 2021; Palmer et al., 2021). This differs from our previously published work that investigated functionally selective compounds utilizing relatively simple cognitive behavioral paradigms, like temporal order memory in an object recognition paradigm (Bransom et al., 2024) or spatial WM in the T-maze (Yang and Mailman, 2018; Yang et al., 2021; Yang et al., 2022b; Cimino et al., 2023). The current study used TUNL task to capture components of multiple cognitive processes simultaneously. The parameters assessed included spatial WM, pattern separation, novelty seeking, sustained attention, and cognitive load which was manipulated by changing stimuli presentation order of unique trial difficulty combinations (e.g., time delay, separation distance) within the same behavioral paradigm. This is advantageous for highlighting differences in simple and complex cognitive processes within the same task and for future investigation of cognitive load by manipulating trial difficulty order (i.e., upfront, gradual, or random trial difficulty sequence). This approach carries significant translational application for drug discovery and assessment, providing a rigorous context in which one can investigate how functionally selective ligands differentially change components of complex behavior. Combined with standardized training and assessment protocols, reduced interhandler variability, and the ability to record a breadth of behavioral parameters (Kangas and Bergman, 2017; Sullivan et al., 2021), the current study design significantly increases the likelihood of reproducibility (Sha et al., 2023) and translatability.

We found distinct differences and similarities between compounds and doses across various parameters quantified during the TUNL task. Subjects treated with either 2MDHX or PF at 10 nmol/kg displayed a significant increase in the number of correction trials attempted compared with vehicle at the session level but no differences in the number of novel trials initiated. This increase in attempted correction trials may be representative of increased motivation and resilience to completing previously incorrect trials or because of decreased attention to the task itself. In contrast, subjects administered the highest dose (10,000 nmol/kg) of either 2MDHX or PF attempted significantly less trials, which, interestingly, was accompanied by a significantly higher quantity of total beam breaks. An increase in total beam breaks typically implies an increase in subject movement, but when coupled with our session engagement results, our results suggest that those subjects are (1) remaining in the center of the chamber and activating both front and back beams with their tail and head, (2) sitting in line with the front beam, or (3) sitting in line with the back beam. Regardless, subjects that were administered higher doses may be experiencing increased motor behavior, such as grooming, paw shifting, and head bobbing/sniffing (Diaz Heijtz and Castellanos, 2006) that can potentially affect their task engagement. Further characterization of these behaviors is warranted.

Notably, the highest dose of either PF or 2MDHX hindered subjects’ ability to attempt both novel and correction trials, and this was especially severe in PF-treated subjects, such that two subjects treated with a PF dose of 10,000 nmol/kg initiated the start of the task but did not attempt a single trial during the session. On a simple dose basis, 2MDHX was tolerated better than PF in terms of task engagement, consistent with our previous study showing that the optimal dose of 2MDHX to improve temporal order memory tended to be higher than PF (Bransom et al., 2024). This could possibly be due to the differences in signaling bias or to off-target activity of PF versus 2MDHX. Further investigation into functional selectivity and the role of β-arrestin signaling in dose-dependent behavioral outcomes is warranted. It is also possible that subjects treated with higher doses of either compound may have decreased reward expectancy signals (Ott et al., 2023), experience alterations in time perception during the task (Fung et al., 2021), and have increased stationary motor behavior or a combination of these. This is demonstrated by increased standard trial reward collection latency, reduced task engagement, and an increase in the number of total beam breaks, respectively. Additionally, we cannot rule out the effects of these compounds on appetite although our subjects underwent a food deprivation paradigm throughout testing and our selected doses are on the lower end of the spectrum tested for similar compounds, suggesting appetite effects were less involved (Cooper et al., 2006; Katz et al., 2006). Nevertheless, any conclusion should be interpreted cautiously. The current study did not assess the in vivo occupancy of the D_1_ (and D_5_) receptors by each compound at the highest doses, limiting direct dose–dose comparisons.

For memory-related performance accuracy, across the middle range of doses (10, 100, and 1,000 nmol/kg), PF-treated subjects displayed a trend for increase in session accuracy. In contrast, 2MDHX-treated subjects' performance accuracy trend was less clear which may be due to heterogeneous responses where some subjects performed extremely well while others performed poorly at the same dose (Bransom et al., 2024). However, subjects generally had similar or worse performance accuracy than vehicle across the tested dose range for both compounds. These findings contrast with our labs (Yang et al., 2021; Yang et al., 2022b; Cimino et al., 2023; Bransom et al., 2024) and other labs (Arnsten et al., 1994; Zahrt et al., 1997; Vijayraghavan et al., 2007; Arnsten et al., 2017; Wang et al., 2019) previously published findings that demonstrate an increase in cognitive enhancement properties for the middle range doses. This could be due to differences in motivation, attention, appetite, and movement as discussed in a previous section. To uncover potential TUNL task-based parameters that may influence overall session accuracy, we incorporated individual trial difficulty into our model investigating correct trial probability. Our model indicated that current trial delay and the previous trials delay × separation distance were significant fixed effects, suggesting that current and previous trial difficulty play a large role in trial performance accuracy and may have obscured the detectable cognitive enhancement effects of the drugs. Although we did not observe the same cognitive enhancing properties of D_1_ agonists on this task, our results still displayed an inverted U-shaped dose response curve for both compounds which is demonstrated by a trending increase in performance in the middle dose range and reduced task performance at dose extremes (e.g., 10,000 nmol/kg). Further investigation with a narrowed dose range (10–1,000 nmol/kg) and repeated administration will increase our understanding of these compounds functionally selective properties and cognitive enhancing effects.

Most notably, one factor that has not previously been reported on, and that must be considered for this behavioral assay, is time. We incorporated time into our analyses due to the nature of our paradigm which uses a time-based threshold (1 h) rather than a trial attempts quota. Subjects are prompted to initiate and drive participation in this task and are partially in control of the quantity of trials attempted and thereby the amount of novel trial conditions they are exposed to throughout the 60 min session. Our analyses indicated that block, the categorical time variable, was a significant fixed effect for every latency parameter except for correct image response latency for correction trials. Generally, subjects’ image response latency and reward collection time increased in duration across all three blocks in a linear fashion that plateaued in the second or third block. This trending increase in reaction time, combined with a reduction in trials attempted across blocks, suggests novelty-induced cognitive fatigue. Subjects are continuously exposed to unique trial combinations throughout the session (i.e., novelty induced), complete less trials, and have increased reaction time throughout the 1 h session (i.e., cognitive fatigue). Surprisingly, block was not a significant predictor of correct choice probability, indicating that the probability of selecting the correct choice was not significantly different throughout the session. In other words, task engagement was reduced throughout the course of the session independent of individual trial performance. These findings demonstrate that subjects reduce overall participation in the task across blocks, but their WM function remains consistent. This type of cognitive fatigue, which is defined as the mental effort required to successfully perform and remain engaged in a task following exposure to increasing levels of new information, did not affect memory-related performance accuracy. This highlights a potential dissociation between different cognitive processes that could be leveraged in the development of targeted therapeutics.

In conclusion, our findings do not support our original hypotheses that either compound (PF or 2MDHX) would be superior in improving cognitive performance and moreover, that the signaling profiles between PF and 2MDHX would translate into differences in behavioral performance. Instead, the cognitive enhancement and the differences between PF and 2MDHX were both very modest. The results, however, lead to several valuable conclusions about the TUNL task itself as well as the potential use of D_1_ agonists for cognition. Recent clinical trials have investigated the beneficial effect of a partial D_1/5_ agonist in the clinical population. The clinical candidate tavapadon (CVL-751; PF-06649751), like PF-06412562, is highly biased for cAMP signaling with little β-arrestin activation. It clearly improves motor function and increases cognition, motivation, and attention in Parkinson's disease patients (Gray et al., 2018; Sohur et al., 2018; Riesenberg et al., 2020; Young et al., 2020; Lewis et al., 2023; Bezard et al., 2024). It is likely that the non-motor benefits in Parkinson's patients are due more D_1_-mediated normalization of the basal ganglia circuitry than to direct effects on cognitive processes. Whatever the mechanism that benefits PD patients, the translation to treating cognitive deficits caused by aging or other disorders should carefully consider the biology of the target population and their likelihood of a response. The current findings, if translatable, underscore the importance of an optimized dose range for each subject (and presumably, disorder). If this latter hypothesis is correct, it will be challenging in determining how to individualize the dose when there is not a clear way to see benefit (as there is in PD). Addressing this will be important in the investigation of daily, low-dose administration of functionally selective compounds and assessing the effects of long-term administration on learning and memory processes (Puig et al., 2014; De Risi et al., 2025).

## Synthesis

Reviewing Editor: Michael McDannald, Boston College Decisions are customarily a result of the Reviewing Editor and the peer reviewers coming together and discussing their recommendations until a consensus is reached. When revisions are invited, a fact-based synthesis statement explaining their decision and outlining what is needed to prepare a revision will be listed below. The following reviewer(s) agreed to reveal their identity: Jill Silverman. Note: If this manuscript was transferred from JNeurosci and a decision was made to accept the manuscript without peer review, a synthesis may not be available.

Both reviewers find the manuscript interesting and agree that the study provides valuable data, but they also concur that several interpretations require further clarification and support. Both highlight that the main findings show modest or predominantly negative effects, which conflict with the claims of cognitive enhancement and an inverted-U dose-response curve in the abstract and manuscript. These claims thus need to be more closely aligned with the study's findings. A shared major concern is the lack of controls for motor function and other potential confounds (such as age-related performance decline, appetite, and drug washout), which weakens the conclusions about cognitive mechanisms. The reviewers also note that the interpretation of novelty-induced cognitive fatigue is insufficiently supported, and that alternative explanations should be considered. Concerns with data presentation, such as the use of nonstandard data transformations instead of raw data, also need to be addressed. Overall, while the study is potentially valuable, both reviewers recommend major revisions, including the inclusion of additional control data on motor activity, clearer justification of interpretations and additional discussion to support mechanistic claims.

Please find the specific comments and queries raised by the reviewers below:

Reviewer 1:

The introduction of novel cognitive enhancers would be of great benefit to society, generally, beyond caffeine, especially if such compounds were non-addictive and less susceptible to rapid tolerability. Moreover, in expansion, many neuropsychiatric disorders with large populations have intellectual and developmental disabilities in cognition or cognitive decline in neurodegeneration, making this class of compounds desirable, for novel therapeutics. Dopamine D1 receptor (D1R) signaling in the brain has been strongly implicated in multiple cognitive processes, with D1 agonists known to enhance performance. The compounds assessed herein are selective D1 receptor agonists, shown previously to predominantly act via one pathway exclusively 2 MDHX (the dogma cAMP pathway) versus PF-06256142, acting via both the cAMP and the beta-arrestin pathway. D1 activation generally is strongly implicated in multiple cognitive processes, motivational efforts and motor activity. It is also known, because of all the components of D1 activation's functions that D1 agonists typically enhance performance in operant cognitive assays. Herein, the work explored how functionally selective D1 receptor agonists, 2MDHX and PF 06256142 are working as cognitive enhancers, using the operant touchscreen assay of TUNEL, an assay of spatial working memory and pattern separation assay, in rats. The development of functionally selective D1 agonists may offer precision therapy if we understand how signaling bias impacts the multiple components of integrated cognitive processes in complex tasks. Results revealed an inverted U-shaped dose response curve for both compounds, with mid-range doses (10, 100, and 1,000 nmol/kg) showing "improved task performance" and "trial numbers (i.e., motivation)", whereas the highest dose significantly reduced these parameters. The authors conclude that "these findings underscore the importance of dose optimization and task structure, while showcasing the translational value of touchscreen-based paradigms when assessing cognitive enhancers, concomitantly highlighting the therapeutic potential of D1 agonists," a statement which this reviewer fully supports and agrees. Along with the fact that this extensive amount of data is well worth report; however, limitations and cautious interpretations need to be employed, and there were several other points to discuss/clarify precluding me from "accepting" this version "as is."

Major comments:

• A broad limitation was the absence of either motor skills data within the existing task or as an accompanying assay, in addition to control testing that measured simpler cognitive indices, or component delineated metrics, from this assay, which would have strengthened the interpretation provided by the authors, immensely. They examined two selective D1 agonists, 2-methyldihydrexidine (2MDHX) and PF-06256142 (PF), on performance in Trial-Unique Nonmatching-To-Location (TUNL). This is an elegant, difficult and laborious, in vivo pharmacology assay, which the authors' propose will highlight the critical role of dose optimization for a balance of in vivo efficacy and tolerability, and which adds to understanding of how D1R signaling influences working memory, for translational studies, as this behavioral paradigm requires a high cognitive demand to maintain performance, throughout the testing sessions, and is a complex task. While this all may be true, without eliminating or even acknowledging, the simple confounds, the higher-level interpretations are less believable.

• The second major comment that was tough to get over for me was the age of the animals at the start of the testing; they ranged from 5-9 months. This is an enormous range, and I am unsure how much subject rodents will even perform post 6 months, once breeding is in stark decline, and they are turning into older adults from young adults.

• While I agree the task is sophisticated and complex, their unique analysis, and nonstandard reporting of the data, made the findings even harder to understand. Secondly, I found their leap to determine the intracellular signaling mechanism underlying integrated findings, overinterpreted, and lacking in careful and precise results descriptions.

• The authors claim that functional selectivity/bias however did not pose as a major factor in this cognitive task; however, there were no control data that illustrated this fact. They also claim that they observed novelty-induced cognitive fatigue, by increased reaction and reward collection latencies and decreased trial attempts without a decline in choice accuracy, can the authors explain this further? The decreased trial attempts certainly illustrate poor engagement and motivation, but it is unclear how they came to the fact of "novelty induced fatigue"

• The results herein underscore the importance of optimized dose ranges for each compound being assessed and each delineated behavioral domain being measured. This dose range part is supported by the data herein but the delineation of behavioral components is not and is missing from this report.

• In their discussion the authors correctly assess the common queries on the face validity of rodent cognitive assays, specifically, the touchscreen assays. However, given the popularity of the NIH toolbox for cognition, the Cambridge CANTAB for neuropsychological cognitive assessments and the NIH baby toolbox for neurodevelopmental/intellectual disability disorders, I do not see "touchscreen assays" as lacking face validity, and this discussion point seemed unnecessary. However, their argument to get away from simple "ethological based" mouse tasks is a good one, bc often underlying circuitry of mouse tasks is unknown to have human analogs or may be species specific, a major missing component to this argument regarding simple assays is that there are simple aspects of learning and memory and simple behaviors of locomotion and motivation that could be assessed within the touchscreen testing that could have been assessed within this study priori to TUNL and would have aided greatly in the delineation of the simple components (motor abilities, motivation to perform), from the complex components of TUNL cognition, given that the data outcomes indices are measuring multiple integrated variables.

• The authors choice to transform the data over illustrating raw numbers of trials, percent correct/accuracy, survival curves and latency to obtain the reward or begin a trial in seconds was confusing and without a clear justification. Why transform to probability and log scales?

• Subjects administered the highest dose of either 2MDHX, or PF, attempted significantly less trials, had significantly lower session accuracy scores, and had longer reward collection durations compared to vehicle sessions. The highest dose also adversely affected the subjects' ability to attempt both novel and correction trials. This was especially severe in PF treated subjects, such that two subjects treated with a dose of 10,000 nmol/kg initiated the start of the task but did not attempt a single trial during the session. This was interpreted by the authors as "supporting the notion that at higher doses, 2MDHX may be tolerated better than PF in terms of task engagement," however without controls on how the drugs affect motor abilities, at these older ages, and at these high doses, I do not know how they can conclude that interpretation with strong conviction, as I imagine that a high dose may adversely affect motor skills to the point that they "don't move" or "perform" in the task at all, and/or conversely, that the highest dose of either compound may make the subjects so hyperactive that they get the same result This is not to say that their interpretation was wrong, I just do not see supporting data to come to that conclusion, and therefore, it needs elaboration.

• In Figure 4, why is the trial number so variable? Could their be motor effects of the drugs? Also, why is trial number is so low comparatively to other rat pharm studies on TUNEL, couldn't it be age related?

• Another conclusion from the authors was while the findings did not support their original hypothesis that either compound (PF or 2MDHX) would be superior in improving cognitive performance and this would be due to their varying mechanisms of action in cellular signaling. Effects, if seen at all, were very modest and thus they speculate about the potential use of D1 agonists for cognition, however, reported beneficial effects of partial D1 agonists in PD, that are biased for cAMP signaling, illustrate improvements in motor function, motivation, and attention, in PD patients. The authors then speculate that the PD findings are more likely to be the result of normalization of the basal ganglia circuitry than the result of specific effects on cognitive processes, which uses their findings, herein, to extend to other adjacent work, and make quite a big statement, that is not directly supported herein. The authors agree they do not know what mechanism is benefitting PD patients, aging or other disorders, and thus while they encourage careful subsets of patients, with stratification, their caution should also extend to their own preclinical interpretation, given the 100s of failures of AD drugs over the past decade.

Minor comments:

1. The age/weight of the subjects was of too broad of a range and frankly, it is likely the animals were too old to get them to perform enough in this assay. This fact is clearly illustrated by the vehicle and Dose 0 groups not reaching 20 trials.

2. In Subjects description, [Author, University] needs to be corrected two times.

3. Illustrating the days to complete pretraining sessions in a table or graphs would be helpful to prove me wrong that the rats were too old.

4. How do I not know that the mock sessions did not influence the data? How do these mock sessions figure into the number of sessions performed to criteria? Statistical testing illustrating no differences is required.

5. In Figure 2, why are doses 0, 1, 2, and 4, less than vehicle w 2MDX, shouldn't 0=vehicle? Doses 2 and 3 of PF clearly improve accuracy, but again why is 0 not=vehicle? Why are the number of rats differ per group, often by many?

6. Why for one training step was the criteria lowered to 50% over 60% as in all other stages, and why was this criterion lowered than the standardized criteria of the Bussey-Saksida group?

7. More explanation on session data versus trials data is required. Since this analysis is nonstandard, it is unclear why it is being performed and what is shows/highlights for the reader.

Reviewer 2:

This manuscript provides advances in the field of potential cognitive enhancers based on the activation of dopamine receptor pathways. The authors evaluated the performance of adult male rats in the TUNL task using two different D1 agonists with separate intracellular signaling profiles. I believe the manuscript provides valuable information on the action of these compounds in a complex cognitive task that likely involves working memory, perceptual and mnemonic discrimination, decision making and motivation, among other functions. I do, however, have a number of comments to improve the MS and some questions regarding the interpretation of the results.

1- In the abstract, the authors wrote "Our results revealed an inverted U-shaped dose response curve for both compounds, with mid-range doses (10, 100, and 1,000 nmol/kg) displaying trends towards improved task performance and engagement, whereas the highest dose significantly reduced these parameters." I think that, overall, the abstract does not represent the results obtained. From the abstract, I expected to find some cognitive improvement or no effect, but the main results indicate that there is a deficit in the performance of the task for both drugs, almost independently of the dose. I agree that there might be differences between doses, but the overall impression is that the performance is worse that vehicle.

2- Why only male rats were evaluated? Are there any known differences between sexes in the effect of these drugs?

3- In the discussion, the authors wrote "These findings are consistent with our (Yang et al., 2021; Yang et al., 2022a; Cimino et al., 2023; Bransom et al., 2024), and other labs (Arnsten et al., 1994; Zahrt et al., 1997; Vijayraghavan et al., 2007; Arnsten et al., 2017; Wang et al., 2019) previously published findings demonstrating that D1 agonists follow an inverted-u shaped dose-response curve, peaking in cognitive enhancement properties for the middle range doses and reducing task performance at both extremes." I don't think this is accurate. I would consider discussing more thoroughly the differences in performance found between the effect of the drugs in the spatial alternation and the temporal order memory tasks and the effect over TUNL. While these drugs might act as cognitive enhancers in previous experiments, they do not appear to do it in this set of experiments. There are a number of reasons why this could have happened like the distinct demands on motivation and the duration of the tasks and test sessions, among others.

4- The authors also infer that the decrease in trials attempted is due to novelty induced cognitive fatigue. I understand this change is observed in all groups, included the controls. However, there seems to be a marked decrease in trial attempts at high doses of PF. I believe that, although cognitive fatigue could effectively be one reason to explain the results, the authors have not excluded other potential confounders. Could these drugs affect appetite? There are a few reports on dopamine agonists decreasing food intake. Is locomotor activity affected by the compounds? Is 5 days enough time for effective washout of the drugs at any dose injected?

## Author Response

Synthesis of Reviews:

Both reviewers find the manuscript interesting and agree that the study provides valuable data, but they also concur that several interpretations require further clarification and support. Both highlight that the main findings show modest or predominantly negative effects, which conflict with the claims of cognitive enhancement and an inverted-U dose-response curve in the abstract and manuscript. These claims thus need to be more closely aligned with the study's findings.

We agree with your assessment and have revised the manuscript accordingly in the abstract (page 2, lines 47-52) and in the discussion section (page 15, lines 623-628, 635-638) to report claims accurately aligned with the study's findings.

A shared major concern is the lack of controls for motor function and other potential confounds (such as age-related performance decline, appetite, and drug washout), which weakens the conclusions about cognitive mechanisms.

We acknowledge that our manuscript required additional justifications for potential confounds and additional support describing alternative explanations of results from this multifaceted behavioral task. These have all been addressed in the revision through added analyses or discussions. Please see below for more details.

The reviewers also note that the interpretation of novelty-induced cognitive fatigue is insufficiently supported, and that alternative explanations should be considered.

We have provided additional explanations on novelty-induced cognitive fatigue and updated our discussion section to include additional interpretations of our findings (page 14, lines 587-589, 591-598; page 15, lines 605-618; page 16, lines 651-654, 657-662).

Concerns with data presentation, such as the use of nonstandard data transformations instead of raw data, also need to be addressed.

We apologize for the confusion regarding our reported values. We have provided additional explanations in the methods section (page 7, lines 270-273), in the figure and table legends for each analysis, and revised them to report numbers based on raw data. Please see the manuscript and our feedback below for more details.

Overall, while the study is potentially valuable, both reviewers recommend major revisions, including the inclusion of additional control data on motor activity, clearer justification of interpretations and additional discussion to support mechanistic claims.

We thank the reviewer for these valuable suggestions and have addressed them as noted below. We feel that the resulting changes have markedly improved the manuscript, and hope that the reviewers agree.

Reviewer 1:

The introduction of novel cognitive enhancers would be of great benefit to society, generally, beyond caffeine, especially if such compounds were non-addictive and less susceptible to rapid tolerability. Moreover, in expansion, many neuropsychiatric disorders with large populations have intellectual and developmental disabilities in cognition or cognitive decline in neurodegeneration, making this class of compounds desirable, for novel therapeutics. Dopamine D1 receptor (D1R) signaling in the brain has been strongly implicated in multiple cognitive processes, with D1 agonists known to enhance performance. The compounds assessed herein are selective D1 receptor agonists, shown previously to predominantly act via one pathway exclusively 2 MDHX (the dogma cAMP pathway) versus PF-06256142, acting via both the cAMP and the beta-arrestin pathway. D1 activation generally is strongly implicated in multiple cognitive processes, motivational efforts and motor activity. It is also known, because of all the components of D1 activation's functions that D1 agonists typically enhance performance in operant cognitive assays. Herein, the work explored how functionally selective D1 receptor agonists, 2MDHX and PF 06256142 are working as cognitive enhancers, using the operant touchscreen assay of TUNEL, an assay of spatial working memory and pattern separation assay, in rats. The development of functionally selective D1 agonists may offer precision therapy if we understand how signaling bias impacts the multiple components of integrated cognitive processes in complex tasks. Results revealed an inverted U-shaped dose response curve for both compounds, with mid-range doses (10, 100, and 1,000 nmol/kg) showing "improved task performance" and "trial numbers (i.e., motivation)", whereas the highest dose significantly reduced these parameters. The authors conclude that "these findings underscore the importance of dose optimization and task structure, while showcasing the translational value of touchscreen-based paradigms when assessing cognitive enhancers, concomitantly highlighting the therapeutic potential of D1 agonists," a statement which this reviewer fully supports and agrees. Along with the fact that this extensive amount of data is well worth report; however, limitations and cautious interpretations need to be employed, and there were several other points to discuss/clarify precluding me from "accepting" this version "as is." Major comments:

A broad limitation was the absence of either motor skills data within the existing task or as an accompanying assay, in addition to control testing that measured simpler cognitive indices, or component delineated metrics, from this assay, which would have strengthened the interpretation provided by the authors, immensely. They examined two selective D1 agonists, 2-methyldihydrexidine (2MDHX) and PF-06256142 (PF), on performance in Trial-Unique Nonmatching-To-Location (TUNL). This is an elegant, difficult and laborious, in vivo pharmacology assay, which the authors' propose will highlight the critical role of dose optimization for a balance of in vivo efficacy and tolerability, and which adds to understanding of how D1R signaling influences working memory, for translational studies, as this behavioral paradigm requires a high cognitive demand to maintain performance, throughout the testing sessions, and is a complex task. While this all may be true, without eliminating or even acknowledging, the simple confounds, the higher-level interpretations are less believable.

We thank the reviewer for this valuable suggestion and fully agree that the potential effects of dopaminergic drugs on motor function should be carefully considered. In our previously published work, we used simpler cognitive tests such as the T-Maze and temporal order object recognition that are dependent on subject mobility. We tested a dose range of 1 - 1,000 nmol/kg for both 2MDHX and PF and found no observed differences in subject mobility {see doi:10.1007/s00213-023-06440-5; 10.1016/j.neuroscience.2024.05.033; 10.1038/s41380-018-0312-1 for additional reference}. For the TUNL task in our current study, we have run an additional analysis quantifying the total number of front and back beam breaks to investigate the subject's movement in the chamber and have added these results to our manuscript (Table 6). Please see this additional section in the methods (page 8, lines 289-295), results (page 12, lines 466-468; page 13, lines 544-550), and discussion (pages 14, lines 589-598).

Along with this additional analysis, we also conducted a comprehensive re-check of the raw data, data processing procedures, and statistical analyses. We found a few individual Excel cells (8 out of 28,826) representing trial-based difficulty parameters and time stamps were inadvertently left blank when data was transferred from Excel to SPSS and thus resulted in some missing data points from our statistical models. These have now been corrected, and all analyses were rerun. The revised results did not alter the conclusions of the study.

The second major comment that was tough to get over for me was the age of the animals at the start of the testing; they ranged from 5-9 months. This is an enormous range, and I am unsure how much subject rodents will even perform post 6 months, once breeding is in stark decline, and they are turning into older adults from young adults.

We agree that the large age range of our subjects engaged in this study contains a broader range than desired. This is due to prolonged training, our within-subjects experimental design, and 5-day washout period between compound exposure sessions. Importantly, we required subjects to perform 60% or higher to advance from no-drug testing to receive the mock session, a 60% threshold during the mock session to advance to the vehicle session, and 60% or higher on the vehicle session to move on to the drug testing session. This demonstrates subjects were able to comprehend and engage in the task (see page 6, lines 226-228, 233-237).

While I agree the task is sophisticated and complex, their unique analysis, and nonstandard reporting of the data, made the findings even harder to understand.

We apologize for the lack of clarity regarding the data analyses and reporting. Detailed descriptions and clarifications have now been added to the Methods (page 7, lines 256-265) and Results (page 9, lines 345-348). For example, one unique analysis in the current study is to report predicted probability of making a correct choice of each trial. This analysis, in contrast to the traditional analysis of percent correct/accuracy of a test session, serves as an important complement to reveal task-related effects on choice outcome. The justification for this unique analysis and data reporting (in their original scales) has now been clarified (page 7, lines 270-273; page 9, lines 345-351). In addition, please see the methods section under data analysis titled "Based on Session" and "Based on Trial" for a more in-depth description regarding justification of each analysis.

Secondly, I found their leap to determine the intracellular signaling mechanism underlying integrated findings, overinterpreted, and lacking in careful and precise results descriptions.

We acknowledge your point about overinterpretation and have revised our manuscript accordingly (page 15, lines 605-607, 615-618). Now, we have included discussion of potential mechanisms, including biased intracellular signaling, for each behavioral component (e.g., performance accuracy vs response latency vs task engagement) in this sophisticated and complex behavioral paradigm. Please see the revision in the Discussion.

The authors claim that functional selectivity/bias however did not pose as a major factor in this cognitive task; however, there were no control data that illustrated this fact.

One of our goals was to compare two D1 agonists that are partial to full agonists for cAMP and vary in β-arrestin recruitment (2MDHX is a super agonist for β-arrestin recruitment whereas PF has little to no recruitment - similar to dopamine) to see if their difference in pharmacological profiles can lead to divergent effects on components of cognition. We did not observe significant differences and thereafter concluded that functional selectivity may not be a major factor in this task specifically. We have revised to make this statement more accurate and acknowledged that additional investigation is warranted.

They also claim that they observed novelty-induced cognitive fatigue, by increased reaction and reward collection latencies and decreased trial attempts without a decline in choice accuracy, can the authors explain this further? The decreased trial attempts certainly illustrate poor engagement and motivation, but it is unclear how they came to the fact of "novelty induced fatigue" We apologize for the lack of clarity regarding novelty-induced cognitive fatigue. We developed the term "novelty induced cognitive fatigue" because of the structure of TUNL task and the results of our data analyses. We chose the first portion of this term ("novelty induced") because subjects are exposed to unique trial combinations throughout the session and must pass them in order to be exposed to the next novel trial. Thus, the "novelty induced" portion is directly related to the task structure. The second half of the term ("cognitive fatigue") was named as a result of our subjects performing less trials throughout the session, increased reaction time, but no change in choice accuracy, like the reviewer accurately described. In addition, this term was developed in reference to human related terms quantifying cognitive fatigue across high and low cognitive load task {1 0.1007/s00221-018-5427-8; 10.3390/brainsci15060541; 10.1007/s00221-019-05712-x; 10.3390/bs14111086; 10.1371/journal.pone.0080222}. We have now added clarifications in the Discussion (please see updated discussion section - page 16, lines 651-654, 657-662).

The results herein underscore the importance of optimized dose ranges for each compound being assessed and each delineated behavioral domain being measured. This dose range part is supported by the data herein but the delineation of behavioral components is not and is missing from this report.

We acknowledge the necessity of additional interpretation and delineation of behavioral components that may change between unique drug and dose combinations. Many of our compound-based differences in behavior occur at dose extremes (low dose vs. extremely high dose) with very few differences for the middle dose range. This is applied to performance accuracy, response latency, and task engagement. We have now expanded our explanation and interpretation for each of them in the Discussion. Please see the discussion section in general and the specific sections listed for dose range optimization of accuracy (page 15, lines 619-641), task engagement (page 14, lines 584-597; 14-15, lines 599-602), and response latencies (page 15, lines 607-616).

In their discussion the authors correctly assess the common queries on the face validity of rodent cognitive assays, specifically, the touchscreen assays. However, given the popularity of the NIH toolbox for cognition, the Cambridge CANTAB for neuropsychological cognitive assessments and the NIH baby toolbox for neurodevelopmental/intellectual disability disorders, I do not see "touchscreen assays" as lacking face validity, and this discussion point seemed unnecessary. However, their argument to get away from simple "ethological based" mouse tasks is a good one, bc often underlying circuitry of mouse tasks is unknown to have human analogs or may be species specific, a major missing component to this argument regarding simple assays is that there are simple aspects of learning and memory and simple behaviors of locomotion and motivation that could be assessed within the touchscreen testing that could have been assessed within this study priori to TUNL and would have aided greatly in the delineation of the simple components (motor abilities, motivation to perform), from the complex components of TUNL cognition, given that the data outcomes indices are measuring multiple integrated variables.

Thank you for this comment and we agree with your sentiment. One of our primary goals was to convey that this task HAS high face validity and we are in complete agreement with your assessment. To address this, we have reworded a portion of the discussion section (page 14, lines 561-563) to better describe our original intent. We have also addressed potential drug effects on simple behaviors (e.g. locomotion) by conducting an additional analysis on beam break that reflects animal's movement in the touchscreen chamber.

The authors choice to transform the data over illustrating raw numbers of trials, percent correct/accuracy, survival curves and latency to obtain the reward or begin a trial in seconds was confusing and without a clear justification. Why transform to probability and log scales? We apologize for the general confusion regarding how our values were reported. We used GLMMs with the appropriate link function and distribution, and the data were analyzed in their original (raw) format without transformation. We reported model-estimated pairwise contrasts and SEs. We have now made sure to report numbers of trials, percent correct/accuracy, and latency to obtain the reward or begin a trial based on raw data (see page 7, lines 270-273 and table legends). The probability of selecting the correct choice on each trial was derived from our statistical modeling of the trials data and reported in its original scale. We reported probability from trial data because we were able to incorporate individual trial difficulty into our assessment of trial accuracy because this is not possible for session accuracy data. This analysis serves as an important complement to the analysis on percent correct/accuracy. These have all been clarified in the revision.

Subjects administered the highest dose of either 2MDHX, or PF, attempted significantly less trials, had significantly lower session accuracy scores, and had longer reward collection durations compared to vehicle sessions. The highest dose also adversely affected the subjects' ability to attempt both novel and correction trials. This was especially severe in PF treated subjects, such that two subjects treated with a dose of 10,000 nmol/kg initiated the start of the task but did not attempt a single trial during the session. This was interpreted by the authors as "supporting the notion that at higher doses, 2MDHX may be tolerated better than PF in terms of task engagement," however without controls on how the drugs affect motor abilities, at these older ages, and at these high doses, I do not know how they can conclude that interpretation with strong conviction, as I imagine that a high dose may adversely affect motor skills to the point that they "don't move" or "perform" in the task at all, and/or conversely, that the highest dose of either compound may make the subjects so hyperactive that they get the same result. This is not to say that their interpretation was wrong, I just do not see supporting data to come to that conclusion, and therefore, it needs elaboration.

As stated in a different comment above, we agree that this concern regarding motor function is valid and must be addressed. We have run an additional analysis quantifying the total number of front and back beam breaks to investigate the subject's movement in the chamber that does not require a subject to start a trial and have added these sections to our manuscript. Please see this additional section in the methods (page 8, lines 289-295), results (page 12, lines 466-468; page 13, 544-550), and discussion (page 14, lines 589-598).

In Figure 4, why is the trial number so variable? Could their be motor effects of the drugs? Also, why is trial number is so low comparatively to other rat pharm studies on TUNEL, couldn't it be age related? Thank you for this comment as we are heavily invested in elucidating this result as well. The trial number may be more variable compared to other rat pharm studies using the TUNL task because we are using a version that does both horizontal and vertical separation patterns (5x3) instead of 7x2, 7x1, or 5x1, making the trial type highly variable and thereby more difficult.

Please see the section above regarding our analysis of compound-based effects on motor skills and age-based performance.

In addition, we did not have a "minimum" trial threshold and instead used the 60-minute duration threshold. As a result, subjects are not held to an arbitrary minimum trial threshold which could also be a contributing factor towards the lower quantity of trials attempted. Additionally, some published papers utilizing this task don't even list the quantity of trials completed.

Another conclusion from the authors was while the findings did not support their original hypothesis that either compound (PF or 2MDHX) would be superior in improving cognitive performance and this would be due to their varying mechanisms of action in cellular signaling. Effects, if seen at all, were very modest and thus they speculate about the potential use of D1 agonists for cognition, however, reported beneficial effects of partial D1 agonists in PD, that are biased for cAMP signaling, illustrate improvements in motor function, motivation, and attention, in PD patients. The authors then speculate that the PD findings are more likely to be the result of normalization of the basal ganglia circuitry than the result of specific effects on cognitive processes, which uses their findings, herein, to extend to other adjacent work, and make quite a big statement, that is not directly supported herein. The authors agree they do not know what mechanism is benefitting PD patients, aging or other disorders, and thus while they encourage careful subsets of patients, with stratification, their caution should also extend to their own preclinical interpretation, given the 100s of failures of AD drugs over the past decade.

We thank the reviewer for this important comment and fully agree that preclinical findings must be interpreted with caution, particularly in light of the numerous unsuccessful therapeutic developments in Alzheimer's disease over the past decade. We have revised in the abstract (page 2, lines 47-52) and in the discussion section (page 15, lines 619-628, 631-641; page 16, lines 674-682) to make accurate conclusions and more cautious interpretations.

Minor comments:

The age/weight of the subjects was of too broad of a range and frankly, it is likely the animals were too old to get them to perform enough in this assay. This fact is clearly illustrated by the vehicle and Dose 0 groups not reaching 20 trials.

As stated above, we agree that the large age range of our subjects engaged in this task contains a broader range than desired, but these subjects were able to comprehend and engage in the task. This is demonstrated by their ability to pass 60% correct choice. In addition, since we used the 5x3 grid and 2 delay times (0.5s and 5.0s), our paradigm version may be more difficult than those currently published which may explain the lower quantity of trials initiated in general.

In Subjects description, [Author, University] needs to be corrected two times.

Thank you, both instances have been updated (page 4, lines 120-124). This was originally included as part of the recommended eNeuro formatting for double blind review process.

Illustrating the days to complete pretraining sessions in a table or graphs would be helpful to prove me wrong that the rats were too old.

Please see the table below that contains our training data duration. The column labeled as "Manual Recommended" displays how many days it usually takes for a rat to pass each training stage. This is based on the companies recommended training guide and published literature in which the same touchscreen chamber and TUNL task program were used to conduct rat experiments. Our training data are either within or close to these recommendations, demonstrating that our subjects were able to learn the task.

How do I not know that the mock sessions did not influence the data? How do these mock sessions figure into the number of sessions performed to criteria? Statistical testing illustrating no differences is required.

This is a valid point regarding differences in mock and vehicle performance. Please see the figure below. We investigated if there was a difference in overall session accuracy between mock and vehicle groups (F(1, 236) = 1.344, p = 0.247) and found no significant difference in session performance accuracy (β =-1.457 , SE = 1.257, t(236) = -1.159, p = 0.247) using the same modeling method described in the methods section of the manuscript.

In Figure 2, why are doses 0, 1, 2, and 4, less than vehicle w 2MDX, shouldn't 0=vehicle? Doses 2 and 3 of PF clearly improve accuracy, but again why is 0 not=vehicle? Why are the number of rats differ per group, often by many? We apologize for the initial confusion. Compound dosages are labeled using a log scale on the x-axis which means compound dosages of 0, 1, 2, and 4 are 1, 10, 100, and 10,000 nmol/kg respectively. Please see the linear values of compound doses displayed in all of our tables to reduce potential confusion.

Subject quantity remains relatively consistent for each unique drug and dose combination, but not all subjects were able to complete each unique drug and dose combination (page 4, lines 108-114). As a result, there was some variation in subject quantity (see Table 1). Also, some of the subject's data points are on the x-axis in Figure 2a which may make counting the number of subjects in each plot more difficult.

Why for one training step was the criteria lowered to 50% over 60% as in all other stages, and why was this criterion lowered than the standardized criteria of the Bussey-Saksida group? Thank you for this point of clarification. The 50% criterion was used in the first training step to familiarize rats with the TUNL task paradigm. This step included only the easiest trial type (the greatest separation distance and shortest delay). It immediately followed shaping, during which rats were gradually trained to interact with the touchscreen and learn the basic operant contingencies, such as touching stimuli to receive a reward. We used a lower criterion (50% instead of 60%) to ease the transition from shaping to performing the full TUNL task, in which rats would be exposed not only to this easiest configuration but also to more difficult trial versions.

More explanation on session data versus trials data is required. Since this analysis is nonstandard, it is unclear why it is being performed and what is shows/highlights for the reader.

Thank you, we agree that our nonstandard analysis of the data requires additional explanation, which has now been added into the revision (page 7, lines 256-265). Our analyses of the session data, such as trial attempted and trial completed, provided important information on how drugs affected task engagement. To best capture how 2MDHX and PF changed task engagement throughout the entire session, our overall session data is divided into three arbitrary time blocks. Session data, however, provided only partial information about task accuracy. To best evaluate the drugs' effects on task accuracy, we incorporated each trial's configuration (see methods section) into the analyses and thereafter used the trials data for these analyses. We believe that each trial's configuration can influence task accuracy. To best isolate drug-related changes in task accuracy, it was necessary to incorporate each trial's configuration into the analyses and to use trials data in addition to session data.

Reviewer 2:

This manuscript provides advances in the field of potential cognitive enhancers based on the activation of dopamine receptor pathways. The authors evaluated the performance of adult male rats in the TUNL task using two different D1 agonists with separate intracellular signaling profiles. I believe the manuscript provides valuable information on the action of these compounds in a complex cognitive task that likely involves working memory, perceptual and mnemonic discrimination, decision making and motivation, among other functions. I do, however, have a number of comments to improve the MS and some questions regarding the interpretation of the results.

In the abstract, the authors wrote "Our results revealed an inverted U-shaped dose response curve for both compounds, with mid-range doses (10, 100, and 1,000 nmol/kg) displaying trends towards improved task performance and engagement, whereas the highest dose significantly reduced these parameters." I think that, overall, the abstract does not represent the results obtained. From the abstract, I expected to find some cognitive improvement or no effect, but the main results indicate that there is a deficit in the performance of the task for both drugs, almost independently of the dose. I agree that there might be differences between doses, but the overall impression is that the performance is worse that vehicle.

Thank you, we agree with your overall assessment of our findings that 2MDHX and PF treated subjects generally have lower or similar performance accuracy compared to vehicle and our initial description of results did not accurately capture that sentiment. We have changed this in the abstract (page 2, lines 47-52) and in the discussion section (page 15, lines 619-628, 635-641). Although our previous findings indicated that D1 agonists generally improve simple cognitive performance, the current study, using a sophisticated and complex behavioral paradigm, found a modest effect for cognitive enhancement for subjects treated with PF for the middle dose range.

Why only male rats were evaluated? Are there any known differences between sexes in the effect of these drugs? Due to the prolonged period required for rats to learn the task and complete all drug and dose combination tests, this study souly focuses on male rats. In female rats, multiple estrous cycles could introduce confounding factors, complicating interpretation of drug effects on task performance. To our knowledge, there are no studies investigating sex differences for these compounds and our next goal is to reproduce this data in female rats (pre and post sexual maturity).

In the discussion, the authors wrote "These findings are consistent with our (Yang et al., 2021; Yang et al., 2022a; Cimino et al., 2023; Bransom et al., 2024), and other labs (Arnsten et al., 1994; Zahrt et al., 1997; Vijayraghavan et al., 2007; Arnsten et al., 2017; Wang et al., 2019) previously published findings demonstrating that D1 agonists follow an inverted-u shaped dose-response curve, peaking in cognitive enhancement properties for the middle range doses and reducing task performance at both extremes." I don't think this is accurate. I would consider discussing more thoroughly the differences in performance found between the effect of the drugs in the spatial alternation and the temporal order memory tasks and the effect over TUNL. While these drugs might act as cognitive enhancers in previous experiments, they do not appear to do it in this set of experiments. There are a number of reasons why this could have happened like the distinct demands on motivation and the duration of the tasks and test sessions, among others.

As stated above (the response to the comment on the Abstract), we agree that the Discussion also needed revision to accurately support our current findings. Many of our compound-based differences in behavior occur at dose extremes (low dose vs. extremely high dose) with very few differences for the middle dose range. In this aspect, our current findings are consistent with prior work showing D1 agonists dose-response curve. The current study, using a sophisticated and complex behavioral paradigm, however, only found a modest effect for cognitive enhancement for subjects treated with PF for the middle dose range, differing from prior work where simple behavioral tests like T-maze and temporal order object reorganization were used. We have now added statements acknowledging these differences and discussed potential reasons causing these inconsistences, including motivation, attention, movement, and others (page 14, lines 587-598; page 15, lines 605-618, 628-638).

The authors also infer that the decrease in trials attempted is due to novelty induced cognitive fatigue. I understand this change is observed in all groups, included the controls. However, there seems to be a marked decrease in trial attempts at high doses of PF. I believe that, although cognitive fatigue could effectively be one reason to explain the results, the authors have not excluded other potential confounders. Could these drugs affect appetite? There are a few reports on dopamine agonists decreasing food intake.

This is a valid concern and D1 agonists at high dosages do tend to decrease food intake. There is potential for the higher doses (1,000 and 10,000 nmol/kg) to impact food intake and this has been addressed in the discussion (page 15, lines 612-615). Both compounds used in this study, to our understanding, have not been investigated for their impact on appetite; however, our doses used are on the lower end of the spectrum tested for similar compounds (e.g. SKF 385393) {DOI: 10.1097/01.fbp.0000205015.67079.f7; 10.1016/j.ejphar.2005.11.046}. In addition, as described in the methods, we employed a chronic food deprivation paradigm throughout testing. We observed little to no food remaining following each day of training, indicating that the subject is consuming all their standard chow. Unfortunately, this does not demonstrate that our compounds reduce appetite during the behavioral paradigm but rather provides insight regarding general food motivation and consumption to a less palatable food source (standard chow vs. chocolate sucrose pellets used in TUNL task). Additional investigation of our chosen compounds effect on appetite is warranted.

Is locomotor activity affected by the compounds? The first reviewer had the same concern, and we have provided answers regarding locomotion above. In brief, our previous work and literature suggest subject mobility will not affect locomotion {see doi:10.1007/s00213-023-06440-5; 10.1016/j.neuroscience.2024.05.033; 10.1038/s41380-018-0312-1 for additional reference}. However, for this task, we ran an additional analysis quantifying the total number of front and back beam breaks to investigate the subject's movement in the chamber. Please see this additional section in the methods (page 8, lines 289-295), results (page 12, lines 466-468; page 13, 544-550), and discussion (pages 14, lines 589-598).

Is 5 days enough time for effective washout of the drugs at any dose injected? Based on these compounds' half-lives {PMID30431269; PMC6741759}, a 5-day washout period is more than enough time to clear the compound from the subject's system and return to baseline prior to administration of the next combination. In addition, the 5-day washout period was the minimum amount of time between two drug test sessions
